# On the Determination of Lagrange Multipliers for a Weighted LASSO Problem Using Geometric and Convex Analysis Techniques

**DOI:** 10.1007/s00245-023-10096-0

**Published:** 2024-01-17

**Authors:** Gianluca Giacchi, Bastien Milani, Benedetta Franceschiello

**Affiliations:** 1 Università di Bologna, Dipartimento di Matematica, Piazza di Porta San Donato 5, 40126 Bologna, Italy; 2 Institute of Systems Engineering, School of Engineering, HES-SO Valais-Wallis, Rue de l’Industrie 23, 1950 Sion, Switzerland; 3 Lausanne University Hospital and University of Lausanne, Lausanne, Department of Diagnostic and Interventional Radiology, Rue du Bugnon 46, Lausanne, 1011 Switzerland; 4 The Sense Innovation and Research Center, Avenue de Provence 82 1007, Lausanne and Ch. de l’Agasse 5, 1950 Sion, Switzerland

**Keywords:** Tuning parameters, LASSO, Convex optimization, Lagrange duality, MRI, Compressed Sensing, 15A29, 47N10, 47A52, 49K99, 49N45, 65F20, 65F22, 92C55

## Abstract

Compressed Sensing (CS) encompasses a broad array of theoretical and applied techniques for recovering signals, given partial knowledge of their coefficients, cf. Candés (C. R. Acad. Sci. Paris, Ser. I **346**, 589–592 (2008)), Candés et al. (IEEE Trans. Inf. Theo (2006)), Donoho (IEEE Trans. Inf. Theo. **52**(4), (2006)), Donoho et al. (IEEE Trans. Inf. Theo. **52**(1), (2006)). Its applications span various fields, including mathematics, physics, engineering, and several medical sciences, cf. Adcock and Hansen (Compressive Imaging: Structure, Sampling, Learning, p. 2021), Berk et al. (2019 13th International conference on Sampling Theory and Applications (SampTA) pp. 1-5. IEEE (2019)), Brady et al. (Opt. Express **17**(15), 13040–13049 (2009)), Chan (Terahertz imaging with compressive sensing. Rice University, USA (2010)), Correa et al. (2014 IEEE International Conference on Acoustics, Speech and Signal Processing (ICASSP) pp. 7789–7793 (2014, May) IEEE), Gao et al. (Nature **516**(7529), 74–77 (2014)), Liu and Kang (Opt. Express **18**(21), 22010–22019 (2010)), McEwen and Wiaux (Mon. Notices Royal Astron. Soc. **413**(2), 1318–1332 (2011)), Marim et al. (Opt. Lett. **35**(6), 871–873 (2010)), Yu and Wang (Phys. Med. Biol. **54**(9), 2791 (2009)), Yu and Wang (Phys. Med. Biol. **54**(9), 2791 (2009)). Motivated by our interest in the mathematics behind Magnetic Resonance Imaging (MRI) and CS, we employ convex analysis techniques to analytically determine equivalents of Lagrange multipliers for optimization problems with inequality constraints, specifically a weighted LASSO with voxel-wise weighting. We investigate this problem under assumptions on the fidelity term $\left\Vert Ax-b\right\Vert _2^2$, either concerning the sign of its gradient or orthogonality-like conditions of its matrix. To be more precise, we either require the sign of each coordinate of $2(Ax-b)^TA$ to be fixed within a rectangular neighborhood of the origin, with the side lengths of the rectangle dependent on the constraints, or we assume $A^TA$ to be diagonal. The objective of this work is to explore the relationship between Lagrange multipliers and the constraints of a weighted variant of LASSO, specifically in the mentioned cases where this relationship can be computed explicitly. As they scale the regularization terms of the weighted LASSO, Lagrange multipliers serve as tuning parameters for the weighted LASSO, prompting the question of their potential effective use as tuning parameters in applications like MR image reconstruction and denoising. This work represents an initial step in this direction.

## Introduction

Basis Pursuit is a well-known convex minimization problem that was first introduced by F. Santosa and W. W. Symes in 1986, cf. [50], in its simplest formulation:

1 $$\begin{aligned} \text {minimize} \quad \left\Vert x\right\Vert _1+\lambda \left\Vert Ax-b\right\Vert _2^2, \qquad x\in \mathbb {R}^{n}, \end{aligned}$$

where $A\in \mathbb {R}^{m\times n}$, the so-called *design matrix*, and $b\in \mathbb {R}^m$ are fixed. The same problem was later applied to signal processing by S. S. Chen and D. Donoho in 1994, cf. [15]. In 1996, R. Tibshirani re-introduced it as linear regression method, under the name of LASSO. Namely, in [52], they consider the constrained minimization problem:

2 $$\begin{aligned} \text {minimize}\quad \left\Vert Ax-b\right\Vert _2^2, \qquad x\in \mathbb {R}^n, \ \left\Vert x\right\Vert _1\le \tau , \end{aligned}$$

for $$\tau >0$$ and

$$\begin{aligned} \left\Vert x\right\Vert _1:=\sum _{j=1}^n|x_j| \end{aligned}$$

is the $\ell _1$ norm. We will discuss the equivalence between (1) and (2) in the following.

Mathematical analysis approaches to study LASSO problems in all their facets are not new, and the literature is so vast that we can only limit ourselves to mention a few examples, cf. [5, 16, 18, 38, 42, 43, 51]. For instance, in [53, 54], the authors study representation theorems for the solutions of general problems:

$$\begin{aligned} \arg \min _{x}E(b,\nu (x))+\gamma (\left\Vert x\right\Vert ), \end{aligned}$$

in the framework of Banach space theory, where *E* is a loss functional, $\nu$ is a so-called *measurement mapping*, $\gamma$ is a strictly increasing convex function and $\left\Vert \cdot \right\Vert$ is a Banach norm, we refer to [53, Theorem 2], [54, Theorem 2, Theorem 3] for more precise statements. In [3], the authors use convex analysis and variational calculus to study regularity properties of the set-valued mapping:

$$\begin{aligned} (b,\lambda )\in \mathbb {R}^m\times (0,+\infty )\mapsto \arg \min _{x\in \mathbb {R}^n}\frac{1}{2}\left\Vert Ax-b\right\Vert _2^2+\lambda \left\Vert x\right\Vert _1. \end{aligned}$$

The main purpose of this work is to shed new light on the analytic dependence between the *Lagrange multipliers*, understood as explained below, and the constraints of a specific version of a constrained *generalized LASSO* problem. In this version, the relationship can be explicitly computed under further assumptions on target function. Let us provide a detailed explanation and motivation for this interest. As aforementioned, in its simplest definition, LASSO consists in the minimization of the function:

3 $$\begin{aligned} \left\Vert Ax-b\right\Vert _2^2+\lambda \left\Vert x\right\Vert _1, \end{aligned}$$

where $A\in \mathbb {R}^{m\times n}$ and $b\in \mathbb {R}^m$ is a measurement vector. Clearly, problems (1) and (3) have the same minimizers and, therefore, for the purposes of this work, we will consider them as the same minimization problem. In short, (1) and (3) can be interpreted as regularization problems, where the aim is to minimize simultaneously the fidelity term $\left\Vert Ax-b\right\Vert _2^2$, that measures noise, and the regularization term $\left\Vert x\right\Vert _1$, that enforces sparsity. Recall that a vector $x=(x_1,\ldots ,x_n)$ is $s-$**sparse** if $\text {card}\{j : x_j\ne 0\}\le s$. When *s* is clear from the context or irrelevant, we drop *s* and say that *x* is *sparse*. In several applications, *x* is not sparse itself, but it is sparse with respect to a so-called *sparsity-promoting transform* $\Phi :\mathbb {R}^n\rightarrow \mathbb {R}^N$. Stated differently, when $\Phi x$ is known to be sparse, problem (3) can be generalized to:

4 $$\begin{aligned} \text {minimize}\quad \left\Vert Ax-b\right\Vert _2^2+\lambda \left\Vert \Phi x\right\Vert _1, \qquad x\in \mathbb {R}^n, \end{aligned}$$

i.e. the regularization term $\left\Vert x\right\Vert _1$ in (3) is replaced by $\left\Vert \Phi x\right\Vert _1$. The parameter $\lambda >0$ in (3) acts as a tuning parameter that balances the contributions of the fidelity term $\left\Vert Ax-b\right\Vert _2^2$ and regularization addendum $\left\Vert \Phi x\right\Vert _1$: small values of $\lambda$ lower the contribution of the regularization, strengthening the effect of the fidelity term; vice-versa, large values of $\lambda$ make $\left\Vert Ax-b\right\Vert _2^2$ negligible and force $\left\Vert \Phi x\right\Vert _1$ to be small in order for the overall sum to be small. Consequently, solutions corresponding to $\lambda \ll 1$ will be noisy, being close to the set $A^{-1}b$, while solutions $x^\#$ corresponding to $\lambda \gg 1$ have $\Phi x^\#$ more sparse. From this perspective, estimates of tuning parameters for inverse problems can be performed pursuing different approaches. *A posteriori rules* can be used when some a-priori knowledge on the amplitude of noise $e\in \mathbb {R}^m$ is available, say $\left\Vert e\right\Vert _2\le \varepsilon$. For instance, using Morozov’s discrepancy principle, $\lambda$ can be chosen so that a solution $x_\lambda$ of (3) satisfies $\left\Vert Ax_\lambda -b\right\Vert _2\le \varepsilon$, cf. [9, 29, 33]. *A priori rules* require knowledge of noise level, as before, but also a-priori information on the regularity of the solution. For this reason, a-priori approaches are usually bad suited for applications, cf. [2]. Heuristic methods, such as the L-curve are also available, cf. [11, 28, 34]. The L-curve method consists of choosing the optimal tuning parameter empirically by tracing a trade-off curve (the L-curve), whereas the generalized cross-validation (GCV) is a well-performing method that requires high-dimensional matrix calculus, cf. [26, 31, 55]. Other non-standard methods can be found in [32, 45], where the parameter is chosen so that statistical properties of noise, such as whiteness, are optimized; an implementation that avoids the computation of matrix inverses can be found in [6]. CNN and other learning methods were deployed in [30, 41], while a more statistical point of view was adopted in [10].

However, the very reason why $\lambda$ is interpreted as a trade-off between noise and sparsity, in (1) and (3), is that it depends on estimates that are usually unavailable, such as a-priori upper bounds for the $\ell _1$ norm of the unknown vector, i.e. a-priori information on the sparsity of the solution, or upper bounds for the noise, cf. [42]. For $A\in \mathbb {R}^{m\times n}$, $b\in \mathbb {R}^m$ and $\eta \ge 0$, the function:

5 $$\begin{aligned} L(x,\lambda )=\left\Vert x\right\Vert _1+\lambda (\left\Vert Ax-b\right\Vert _2^2-\eta ^2) \end{aligned}$$

is the Lagrangian associated to the constrained minimization problem:

6 $$\begin{aligned} \text {minimize} \quad \left\Vert x\right\Vert _1, \qquad x\in \mathbb {R}^n, \left\Vert Ax-b\right\Vert _2^2\le \eta ^2, \end{aligned}$$

cf. [7]. Roughly speaking, this entails that (1) and (6) are equivalent, up to choosing:

7 $$\begin{aligned} \lambda =\lambda (\eta ) \end{aligned}$$

or, equivalently, $\eta =\eta (\lambda )$, properly. Please note that $\eta$ may not be uniquely determined. We refer to [23, Proposition 3.2] for a more precise statement of this fact. Throughout this work, we call the parameter $\lambda$ in (7) a *Lagrange multiplier* associated to (6), since it plays the same role of Lagrange multipliers in optimization problems with equality constraints. We will use this terminology in a more general setting, see Definition 2.7 below. Since a slightly modified proof of [23, Theorem 3.1] shows that a solution of (6), if unique, must be *m*-sparse, the $\ell _1$ norm is said to *enforce sparsity*. For this reason, the Lagrange multipliers in (5) could be used, in principle, in an equivalent manner as *tuning parameters* for (1) to recover sparse vectors.

In the same way,

8 $$\begin{aligned} L(x,\lambda )=\left\Vert Ax-b\right\Vert _2^2+\lambda (\left\Vert \Phi x\right\Vert _1-\tau ) \end{aligned}$$

is the Lagrange function of the constrained problem:

9 $$\begin{aligned} \text {minimize} \qquad \left\Vert Ax-b\right\Vert _2^2, \qquad x\in \mathbb {R}^n, \left\Vert \Phi x\right\Vert _1\le \tau . \end{aligned}$$

A first question that may be addressed is whether the corresponding Lagrange multiplier $\lambda$ of (9) could still be used as a tuning parameter in (4). If so, the relationship between the Lagrange multipliers and the constraints of the corresponding constrained problems could be useful in concrete applications, such as static and dynamic MRI, cf. [19, 24]. In MRI, indeed, vectors of interest are MR images, which tend to be approximately sparse with respect to the discrete Fourier transform (DFT), the discrete cosine transform (DCT) or the discrete wavelet transform (DWT), cf. [36]. This means that a solution of the generalized LASSO problem (4), where the design matrix *A* is a proxy of the acquisition methods properties (coil sensitivity, undersampling schemes and DFT), and *b* is an underdetermined, noisy measurement, will have a sparse regularization term, i.e. sparse $\Phi x$. We stress that (4) is known to admit a, in general not unique, solution for any choice of *A*, *b*, $\lambda$ and $\Phi$, and for the sake of completeness we report a proof, that uses only linear algebra, in the appendix. In order to exploit more a-priori knowledge on the structure of MRI data, (4) can be generalized further to consider target functions that are sum of more regularizing terms, cf. [22, 27, 44].

Let us note that sparsity is not always the correct assumption in MRI. For instance, dynamic MR images (e.g. a sequence of images of a moving organ, cf. [19, 24]) are highly compressible, rather than sparse, cf. [37]. This means that most of their coefficients with respect to some sparsity-promoting transform do not vanish, yet are small or negligible.

Surprisingly, it is easier to identify an equivalent of the relationship (7), between the parameter $\lambda$ and the upper bound for the constraint, say $\eta$, when another weighted version of LASSO is considered. Namely, we aim to utilize convex analysis to compute the Lagrange multipliers for the constrained optimization problem:

10 $$\begin{aligned} \text {minimize} \quad \left\Vert Ax-b\right\Vert _2^2, \qquad x\in \mathbb {R}^n, \quad |x_j|\le \tau _j, \quad j=1,\ldots ,n. \end{aligned}$$

For given $\tau _1,\ldots ,\tau _n>0$ and a given minimizer $x^\#$ of (10) there exist $\lambda _1,\ldots ,\lambda _n\ge 0$ such that $x^\#$ is also a minimizer of:

11 $$\begin{aligned} \text {minimize}\quad \left\Vert Ax-b\right\Vert _2^2+\sum _{j=1}^n\lambda _j|x_j|, \end{aligned}$$

see [7, Section 5.3.2] or Theorem 2.4 below for a complete statement. Other weighted versions of this problem have been considered in the literature. For instance, in [41], the authors present a total variation (TV) regularization-based weighted LASSO for image denoising. Other references include [9], where the authors consider *space-variant* problems, such as:

$$\begin{aligned} \text {minimize} \quad \frac{1}{2}\left\Vert Ax-b\right\Vert _2^2+\sum _{j=1}^k\lambda _k\left\Vert (Dx)_j\right\Vert _p, \end{aligned}$$

where $A\in \mathbb {R}^{m\times n}$, $b\in \mathbb {R}^m$, *D* is the discrete gradient, $p\in \{1,2\}$, and $\lambda _1,\ldots ,\lambda _k>0$. In a certain sense, problem (11) can be considered as a space-variant problem, where every component of the unknown vector is weighted by a different parameter. In [46], the author discusses the importance of space-variance in TV regularization, as a mathematical modeling which has the advantage of recovering a description of local features, which is lost by classical TV regularization, i.e. (4) with $\Phi =D$.

As we shall see, the relationship between these parameters is non-trivial if *A* is non orthogonal, due to the complicated geometry of (10). Loosely speaking, this is due to the fact that if $A^TA$ is non-diagonal, *A* shuffles the coordinates of *x* in such a way that each pair of sets $M_j:=\{x\in \mathbb {R}^n: x_j=-\tau _j\}$ and $N_j:=\{x\in \mathbb {R}^n:\frac{\partial }{\partial x_j}(\left\Vert Ax-b\right\Vert _2^2)=0\}$ are no longer parallel.

To summarize, the results contained in this work serve as first steps towards the understanding of the analytical relationship between Lagrange multipliers, as defined in Definition 2.7 below, and tuning parameters for LASSO problems. This relationship is non-trivial, since it involves a-priori estimates, such as estimates of the $\ell _2$-norm of the noise. Our interest is justified by the fact that, plugging the identity as regularization term in (4), the results mentioned above tell that the corresponding Lagrange multipliers yield to the recovery of sparse vectors. Since MR images are known to be sparse or compressible with respect to several sparsify-promoting transforms, the question that arises is whether it is possible to use Lagrange multipliers associated to (9) as tuning parameters to retrieve good-quality anatomical MR images via LASSO problems.

**Overview.** In Sect. 2, we establish preliminaries and notation. In Sect. 3 we compute the deterministic relationships between the parameters $\lambda _j$’s and the $\tau _j$’s in order for problems (10) and (11) to be equivalent, under the following specific assumptions: given *A* such that $A^TA$ is diagonal, for instance when *A* is either a subsampling matrix, the Fourier transform matrix or the identity matrix, the Lagrange multipliers are explicitly given by:

12 $$\begin{aligned} \lambda _j^\#=2\left\Vert a_{*,j}\right\Vert _2^2\left( \frac{|\langle b,a_{*,j}\rangle |}{\left\Vert a_{*,j}\right\Vert _2^2}-\tau _j\right) \chi _{\Big [0,\frac{|\langle b,a_{*,j}\rangle |}{\left\Vert a_{*,j}\right\Vert _2^2}\Big ]}(\tau _j), \end{aligned}$$

where $a_{*,j}$ denotes the *j*-th column of *A* and $\chi _{\Big [0,\frac{|\langle b,a_{*,j}\rangle |}{\left\Vert a_{*,j}\right\Vert _2^2}\Big ]}$ is the characteristic function on $\Big [0,\frac{|\langle b,a_{*,j}\rangle |}{\left\Vert a_{*,j}\right\Vert _2^2}\Big ]$, $j=1,...,n$. We also provide deterministic results for those cases where there is a control on the sign of the gradient of $\left\Vert Ax-b\right\Vert _2^2$, providing the explicit expression of the Lagrange multipliers under the assumption $\frac{\partial }{\partial x_j}(\left\Vert Ax-b\right\Vert _2^2)\le 0$ for every $j=1,\ldots ,n$ in a properly defined hypercube. The conclusions are reported in Sect. 4.

## Preliminaries and notation

**Notation.** For the theory of this section, we refer to [7, 23, 49] as reference therein. We denote by $\mathbb {R}^n$ the *n*-dimensional vector space of real column vectors, whereas $\mathbb {R}^{m\times n}$ denotes the space of real $m\times n$ matrices. To ease the notation, if $x\in \mathbb {R}^n$, the notation $x=(x_1,...,x_n)$ means that *x* is the column vector with coordinates $x_1,...,x_n$. If $A\in \mathbb {R}^{m\times n}$, $A^T$ denotes the transpose of *A*.

If $A\in \mathbb {R}^{m\times n}$, $\ker (A)$ and ${{\,\textrm{Im}\,}}(A)$ denote the kernel and the image of *A*, respectively. $\mathcal {M}_n$ denotes the set of $n\times n$ signature matrices and, for $x\in \mathbb {R}^n$, ${{\,\textrm{sgn}\,}}(x)$ denotes the set of all the possible signatures of *x*, see Sect. 3.2 below.

For $1\le p<\infty$, the $\ell _p$-norm on $\mathbb {R}^n$ is defined as:

$$\begin{aligned} \left\Vert x\right\Vert _p:=\left( \sum _{j=1}^n|x_j|^p\right) ^{1/p}, \qquad x\in \mathbb {R}^n, \end{aligned}$$

whereas $\left\Vert x\right\Vert _\infty :=\max _{j=1,...,n}|x_j|$. We denote by $\langle \cdot ,\cdot \rangle$ the canonical inner product of $\mathbb {R}^n$, i.e.

$$\begin{aligned} \langle x,y\rangle = x^Ty=\sum _{j=1}^nx_jy_j, \qquad x,y\in \mathbb {R}^n. \end{aligned}$$

If $x\in \mathbb {R}^n$, $x^+$ is its positive part, i.e. $x^+\in \mathbb {R}^n$ has coordinates $(x^+)_j=\max \{x_j,0\}$ ($j=1,...,n$). If $\Omega \subseteq \mathbb {R}^n$, $\Omega ^\perp$ denotes its orthogonal complement. For vectors $x,y\in \mathbb {R}^n$, $x=(x_1,\ldots ,x_n)$, $y=(y_1,\ldots ,y_n)$, the notation $x\preceq y$ means that $x_j\le y_j$ for every $j=1,\ldots ,n$. Analogously, $x\prec y$ if $x_j<y_j$ for every $j=1,\ldots ,n$. The relationships $x\succeq y$ and $x\succ y$ are defined similarly.

We always consider $\mathbb {R}^n$ endowed with the Euclidean topology. If $\Omega \subseteq \mathbb {R}^n$, $\mathring{\Omega }$ denotes the interior of $\Omega$ and $\partial \Omega$ denotes the boundary of $\Omega$. If *g* is a real-valued function defined on an open neighbourhood of $x_0\in \mathbb {R}^n$, $\partial g(x_0)$ denotes the subdifferential of *g* at $x_0$, see Sect. 2.2 below for the definition of subdifferential. Using the same notation to denote both the boundary of a set and the subdifferential of a function shall not cause confusion. If $\Omega \subseteq \mathbb {R}^n$, ${{\,\mathrm{a-int}\,}}(\Omega )$ denotes the algebraic interior of $\Omega$, see Definition 3.8 below. If *g* is a function and $\Omega$ is a subset of its domain, $g|_\Omega$ denotes the restriction of *g* to $\Omega$. Finally, if $\Omega \subseteq \mathbb {R}^n$, $\chi _\Omega$ denotes the characteristic function of $\Omega$.

### Lagrange Duality

Consider a constrained optimization problem in the form:

13 $$\begin{aligned} \text {minimize}\, F_0(x), \qquad {\Psi } x=y,\ F_l(x)\le b_l, \ l=1,\ldots ,M, \end{aligned}$$

where $\Psi \in \mathbb {R}^{m\times n}$, $y\in \mathbb {R}^{{m}}$ and $F_0,F_1,\ldots ,F_M:\mathbb {R}^n\rightarrow (-\infty ,+\infty ]$ are convex. We always assume that a minimizer of (13) exists.

A point $x\in \mathbb {R}^n$ is called **feasible** if it belongs to the constraints, that is if:

14 $$\begin{aligned} x\in K:=\Big \{\zeta \in \mathbb {R}^n \ : \ {\Psi }\zeta =y \ and \ F_l(\zeta )\le b_l, \ l=1,\ldots ,M\Big \} \end{aligned}$$

and *K* is called the **set of feasible points**. To avoid triviality, we always assume $K\ne \varnothing$, in which case problem (13) is called **feasible**. In view of the definition of *K*, problem (13) can be implicitly written as:

$$\begin{aligned} \text {minimize}\quad F_0(x), \qquad x\in K. \end{aligned}$$

Convex problems such as (6) and (10) can be approached by considering their Lagrange formulation, see Sect. 2.3 below. The **Lagrange function** related to (13) is the function $L:\mathbb {R}^n\times \mathbb {R}^m\times [0,+\infty )^M\rightarrow (-\infty ,+\infty ]$ defined as:

$$\begin{aligned} L(x,\xi ,\lambda ):=F_0(x)+\langle \xi ,{\Psi } x-y\rangle +\sum _{l=1}^M\lambda _l(F_l(x)-b_l). \end{aligned}$$

Observe that for all $\xi ,\lambda$ and $x\in K$:

$$\begin{aligned} L(x,\xi ,\lambda )=F_0(x)+\underbrace{\langle \xi ,{\Psi } x-y\rangle }_\text {=0}+\sum _{l=1}^M\underbrace{\lambda _l}_{\ge 0} (\underbrace{F_l(x)-b_l}_{\le 0})\le F_0(x), \end{aligned}$$

so that:

15 $$\begin{aligned} \inf _{x\in \mathbb {R}^n}L(x,\xi ,\lambda )\le \inf _{x\in K}L(x,\xi ,\lambda )\le \inf _{x\in K}F_0(x). \end{aligned}$$

#### Definition 2.1

The function $H:\mathbb {R}^m\times [0,+\infty )^M\rightarrow [-\infty ,+\infty ]$ defined as:

$$\begin{aligned} H(\xi ,\lambda ):=\inf _{x\in \mathbb {R}^n}L(x,\xi ,\lambda ) \end{aligned}$$

is called **Lagrange dual function**.

Inequalities (15) read as:

16 $$\begin{aligned} H(\xi ,\lambda )\le \inf _{x\in K}F_0(x) \end{aligned}$$

for all $\xi \in \mathbb {R}^m$ and all $\lambda \in [0,+\infty )^M$. Stating (16) differently, we have the **weak duality inequality**:

W $$\begin{aligned} \sup _{\underset{\lambda \succeq 0}{\xi \in \mathbb {R}^m}}H(\xi ,\lambda )\le \inf _{x\in K}F_0(x). \end{aligned}$$

We point out that (W) is equivalent to:

17 $$\begin{aligned} \sup _{\xi ,\lambda }\inf _xL(x,\xi ,\lambda )\le \inf _x\sup _{\xi ,\lambda }L(x,\xi ,\lambda ) \end{aligned}$$

(see [7, Subsection 5.4.1]).

We are interested in computing the parameters $(\xi ,\lambda )$ such that (W) is an equality, in which case (W) becomes:

S $$\begin{aligned} \sup _{\underset{\lambda \succeq 0}{\xi \in \mathbb {R}^m}}H(\xi ,\lambda )= \inf _{x\in K}F_0(x), \end{aligned}$$

so that **strong duality** (S) holds for problem (13).

### Subdifferential

#### Definition 2.2

**(Subdifferential)** Let $\Omega \subseteq \mathbb {R}^n$ be open and $g:\Omega \rightarrow \mathbb {R}$. Let $x_0\in \Omega$. The **subdifferential** of *g* at $x_0$ is the set:

$$\begin{aligned} \partial g(x_0):=\{v\in \mathbb {R}^n \, \ g(x)\ge g(x_0)+v^T(x-x_0) \ \forall x\in \Omega \}. \end{aligned}$$

We refer to any $v\in \partial g(x_0)$ as a **subgradient** of *g* at $x_0$.

We will use the following proposition.

#### Proposition 2.3

Let $\Omega \subseteq \mathbb {R}^n$ be open and $g:\Omega \rightarrow \mathbb {R}$ be convex and continuous on $\Omega$. Let $x_0\in \Omega$. Then, $\partial g(x_0)\ne \varnothing$.

### Lagrange Formulation of Constrained Problems

Under the notation above, let $F(x):=(F_1(x),...,F_M(x))$. In the convex framework, if the constraint $F(x)\preceq b$ does not reduce to $F(x)=b$, namely if for all $l=1,\ldots ,M$ the inequality $F_l(x)<b_l$ holds for some $x\in \mathbb {R}^n$, then strong duality holds.

#### Theorem 2.4

(Cf. [7], Section 5.3.2) Assume that $F_0,F_1,\ldots ,F_M$ are convex functions defined on $\mathbb {R}^n$. Let $x^\#$ be such that $F_0(x^\#)=\inf _{x\in \mathbb {R}^n}F_0(x)$. If:

(i) there exists ${\tilde{x}}\in \mathbb {R}^n$ such that $\Psi {\tilde{x}}=y$ and $F({\tilde{x}})\prec b$ or,
(ii) in absence of inequality constraints, if $K\ne \varnothing$ (i.e. if there exists ${\tilde{x}}\in \mathbb {R}^n$ such that $\Psi {\tilde{x}}=y$),

then, there exists $(\xi ^\#,\lambda ^\#)\in \mathbb {R}^m\times [0,+\infty )^M$ such that $H(\xi ^\#,\lambda ^\#)=\sup _{\xi ,\lambda }H(\xi ,\lambda )$ and $H(\xi ^\#,\lambda ^\#)=F_0(x^\#)$.

The proof of Theorem 2.4 contains the fundamental construction we will use in the next sections and we report it for this reason. We refer to [7, Subsection 5.3.2] for the complete proof. First, we need a result from functional analysis, which is well-known as (geometrical) *Hahn-Banach theorem*.

#### Definition 2.5

(Separating hyperplane) Consider two subsets $\mathcal {A},\mathcal {B}\subseteq \mathbb {R}^n$. A hyperplane $\Gamma :=\{x\in \mathbb {R}^n \, \ \langle \xi ,x\rangle =\alpha \}$ satisfying:

18 $$\begin{aligned} \langle \xi ,x\rangle \le \alpha <\langle \xi ,y\rangle , \qquad x\in \mathcal {A}, \quad y\in \mathcal {B}, \end{aligned}$$

is a **separating hyperplane** between $\mathcal {A}$ and $\mathcal {B}$.

#### Theorem 2.6

(Cf. [49] Theorem 3.4) Let $\mathcal {A},\mathcal {B}\subset \mathbb {R}^n$ be two convex and disjoint subsets. If $\mathcal {B}$ is open, there exists $\xi \in \mathbb {R}^n$ and $\alpha \in \mathbb {R}$ such that (18) holds for all $x\in \mathcal {A}$ and all $y\in \mathcal {B}$.

#### Idea of the proof of Theorem 2.4

First, one assumes that *A* has full row-rank. Moreover, one reduces to consider the situation in which $p^*:=\inf _{x\in K}F_0(x)>-\infty$, otherwise the assertion is trivial.

Consider the set:

19 $$\begin{aligned} \mathcal {G}:=\Big \{\left( F(x)-b,\Psi x-y,F_0(x)\right) \in \mathbb {R}^M\times \mathbb {R}^m\times \mathbb {R}\ : \ x\in \mathbb {R}^n\Big \}, \end{aligned}$$

where, with an abuse of notation, $\Psi x-y$ denotes the row vector with the same (ordered) entries of $\Psi x-y$, and $\mathcal {A}$ be defined as the epigraph:

20 $$\begin{aligned} \begin{aligned} \mathcal {A}&:=\mathcal {G}+((\mathbb {R}_{\ge 0})^M\times \mathbb {R}^m\times \mathbb {R}_{\ge 0})=\\&=\Big \{(u,v,t)\in \mathbb {R}^M\times \mathbb {R}^m\times \mathbb {R}\ : \ u\succeq F(x)-b, \\&\ \ \ \ \ \ v= \Psi x-y, \ t\ge F_0(x) \ for \ some \ x\in \mathbb {R}^n\Big \}. \end{aligned} \end{aligned}$$

It is easy to verify that if $F_0,F_1,\ldots ,F_M$ are convex, than $\mathcal {A}$ is convex. Then, consider the set:

$$\begin{aligned} \mathcal {B}:=\Big \{(0,0,s)\in \mathbb {R}^M\times \mathbb {R}^m\times \mathbb {R}\, \ s<p^*\Big \}. \end{aligned}$$

$\mathcal {A}$ and $\mathcal {B}$ are clearly disjoint, $\mathcal {B}$ (which is an open half-line) being trivially convex and open. Therefore, the assumptions of Theorem 2.6 are satisfied and we conclude that there exists a triple of parameters $({\tilde{\lambda }},{\tilde{\xi }},\mu )\ne 0$ and $\alpha \in \mathbb {R}$ such that:

21 $$\begin{aligned}&(u,v,t)\in \mathcal {A}\ \ \Longrightarrow \ \ {\tilde{\lambda }}^Tu+\langle {\tilde{\xi }},v\rangle +\mu t\ge \alpha , \end{aligned}$$

22 $$\begin{aligned}&(u,v,t)\in \mathcal {B}\ \ \Longrightarrow \ \ {\tilde{\lambda }}^Tu+\langle {\tilde{\xi }},v\rangle +\mu t<\alpha . \end{aligned}$$

It is easy to see that the definition of $\mathcal {A}$, together with (21), imply that ${\tilde{\lambda }}_l\ge 0$ for all $l=1,\ldots ,M$ and $\mu \ge 0$. Also, applying the definition of $\mathcal {B}$ to (22), one finds that $\mu t<\alpha$ for all $t<p^*$, which implies that $\mu p^*\le \alpha$. Therefore, for all $x\in \mathbb {R}^n$,

23 $$\begin{aligned} \sum _{l=1}^M{\tilde{\lambda }}_l(F_l(x)-b_l)+\langle {\tilde{\xi }},\Psi x-y\rangle +\mu F_0(x)\ge \alpha \ge \mu p^*. \end{aligned}$$

If $\mu >0$, then (23) gives that $L(x,{\tilde{\xi }}/\mu ,{\tilde{\lambda }}/\mu )\ge p^*$ for all $x\in \mathbb {R}^n$, which implies that $H({\tilde{\xi }}/\mu ,{\tilde{\lambda }}/\mu )\ge p^*$. Since the other inequality holds trivially by the weak duality inequality, we conclude that $H({\tilde{\xi }}/\mu ,{\tilde{\lambda }}/\mu )=p^*$. Finally, using the assumptions on the rank of $\Psi$ and on the existence of a point satisfying the strict inequality constraint, one proves by contradiction that it must be $\mu >0$. $\square$

#### Definition 2.7

**(Lagrange Multipliers)** We refer to a couple $(\xi ^\#,\lambda ^\#)\in \mathbb {R}^m\times [0,+\infty )^M$ as to **Lagrange multipliers** for the problem (13) if $(\xi ^\#,\lambda ^\#)$ attend the supremum in (S).

As a consequence of Theorem 2.4, we have the following result, which relates the minimizers of (13) and those of the dual problem $\max _{\xi ,\lambda }H(\xi ,\lambda )$, providing also the Lagrange multipliers, that may not be unique.

#### Corollary 2.8

(Cf. [23] Theorem B.28) Let $F_0:\mathbb {R}^n\rightarrow [0,+\infty )$ and $\phi :[0,+\infty )\rightarrow \mathbb {R}$ be such that $\phi$ is monotonically increasing and $\phi \circ F_0$ is convex. Let $\tau _j>0$ ($j=1,\ldots ,M$) and $\psi _j:\mathbb {R}^n\rightarrow \mathbb {R}$ ($j=1,\ldots ,M$) be convex functions such that $\psi _j^{-1}([0,\tau _j))\ne \varnothing$ for all $j=1,\ldots ,M$. Let $x^\#$ be a minimizer of the problem:

24 $$\begin{aligned} \text {minimize}\quad F_0(x), \qquad x\in \mathbb {R}^n \ \psi (x)\preceq \tau , \end{aligned}$$

where $\tau =(\tau _1,\ldots ,\tau _M)$. Then, there exist $\lambda _j\ge 0$ ($j=1,\ldots ,M$) such that $x^\#$ is a minimizer of:

25 $$\begin{aligned} \text {minimize}\quad \phi (F_0(x))+\sum _{j=1}^M\lambda _j\psi _j(x). \end{aligned}$$

#### Proof

Since $\phi$ is monotonically increasing, (24) is obviously equivalent to:

26 $$\begin{aligned} \text {minimize}\quad \phi (F_0(x)), \qquad x\in \mathbb {R}^n \ \psi _j(x)\le \tau _j, \end{aligned}$$

($j=1,\ldots ,M$) whose Lagrangian is given by:

27 $$\begin{aligned} L(x,\lambda )=\phi (F_0(x))+\sum _{j=1}^M\lambda _j(\psi _j(x)-\tau _j). \end{aligned}$$

By the assumption, $\phi \circ F_0$ and each $\psi _j$ are convex and the inequalities $\psi _j({\tilde{x}})<\tau _j$ are satisfied by some ${\tilde{x}}\in \mathbb {R}^n$ (observe that here we need $\tau _j>0$), so we can apply Theorem 2.4 to get $H(\lambda ^\#)=\phi (F_0(x^\#))$ for some $\lambda ^\#\in [0,+\infty )^M$. By (17), for all $x\in \mathbb {R}^n$:

$$\begin{aligned} L(x^\#,\lambda ^\#)\le L(x,\lambda ^\#), \end{aligned}$$

so that $x^\#$ is also a minimizer of the function $x\in \mathbb {R}^n\mapsto L(x,\lambda ^\#)$. Since the constant terms $-\lambda _j\tau _j$ in (27) do not affect the set of minimizers, we have that $x^\#$ is a minimizer of:

$$\begin{aligned} \text {minimize} \quad \phi (F_0(x))+\sum _{j=1}^M\lambda _j^\#(\psi _j(x)-\tau ), \qquad x\in \mathbb {R}^n. \end{aligned}$$

$$\square $$

#### Remark 2.9

Theorem 2.6 has a complex version that holds with $\Re \langle z,w\rangle =\Re \left( \sum _{j=1}^n\overline{z_j}w_j\right)$ ($\Re$ denotes the real part of a complex number) instead of $\langle \cdot ,\cdot \rangle$. In particular, the entire theory presented in this work is applicable in the complex framework as well. This extension involves replacing the canonical real inner product of $\mathbb {R}^n$ with the real inner product on $\mathbb {C}^n$ defined above. Therefore, we do not need to study the complex case separately, as only the structure of $\mathbb {C}^n$ as a real vector space is involved.

#### Remark 2.10

To sum up, Theorem 2.4 and Corollary 2.8 together tell that, up to the sign, the coefficients of any hyperplane separating the two sets:

$$\begin{aligned} \mathcal {A}=\Big \{(u,t)\in \mathbb {R}^{M+1} \, \ u\succeq F(x)-b, \ t\ge F_0(x) \ for \ x\in \mathbb {R}^n\Big \} \end{aligned}$$

and

$$\begin{aligned} \mathcal {B}=\Big \{(0,t)\in \mathbb {R}^{M+1} \, \ t<\inf _{x\in K}F_0(x)\Big \} \end{aligned}$$

define Lagrange multipliers for problem (13), in absence of equality constraints, i.e. if $y=0$ and $\Psi =0$ in (13). This is the geometric idea that we will apply in the following sections to the weighted LASSO.

## The Weighted LASSO

Let $A\in \mathbb {R}^{m\times n}$, $b\in \mathbb {R}^m$ and $\tau _1,\ldots ,\tau _n\ge 0$. We denote with $a_{*,j}$ the *j*-th column of *A* and set $b=(b_1,\ldots ,b_m)$. We consider the constrained minimization problem:

28 $$\begin{aligned} \text {minimize} \quad \left\Vert Ax-b\right\Vert _2^2, \qquad x\in \mathbb {R}^n, \ |x_j|\le \tau _j, \ {j=1,\ldots ,n}. \end{aligned}$$

We also assume that $\tau _j\ne 0$ for all $j=1,\ldots ,n$. In fact, if $\tau _j=0$ for some $j=1,\ldots ,n$, then the solution $x=(x_1,\ldots ,x_n)$ has $x_j=0$. In this case, problem (28) reduces to

29 $$\begin{aligned} \text {minimize} \quad \left\Vert {\tilde{A}}y- b\right\Vert _2^2,\qquad y\in \mathbb {R}^{n-r}, \ |y_{i_j}|\le \tau _{i_j}, \ {j=1,\ldots ,n-r}, \end{aligned}$$

where $$r=\text {card}\{j : \tau _j=0\}\le m$$, $$J=\{1\le i_1<\ldots <i_{n-r}\le n\}:=\{j : \tau _j\ne 0\}$$ and $${\tilde{A}}=(a_{*,j})_{j\in J}\in \mathbb {R}^{m\times (n-r)}$$.

Let *K* denote the set of the feasible points of problem (28), that is:

30 $$\begin{aligned} K=\{x\in \mathbb {R}^n \ : \ |x_j|\le \tau _j \ \forall j=1,\ldots ,n\} \end{aligned}$$

and consider the Lagrange function associated to (28), i.e.

31 $$\begin{aligned} L(x,\lambda _1,\ldots ,\lambda _n)=\left\Vert Ax-b\right\Vert _2^2+\sum _{j=1}^n\lambda _j(|x_j|-\tau _j). \end{aligned}$$

We are interested in a vector of Lagrange multipliers $\lambda ^\#\succeq 0$ for (28). Based on the proofs of Theorem 2.4 and Corollary 2.8, $\lambda ^\#$ can be chosen as the direction of any hyperplane separating the sets:

32 $$\begin{aligned} \begin{aligned} \mathcal {A}&= \Big \{(u,t)\in \mathbb {R}^n\times \mathbb {R}\ : \ u_l\ge |x_l|-\tau _l \ (l=1,\ldots ,n), \\&\qquad \quad t\ge \left\Vert Ax-b\right\Vert _2^2 \text { for some } x\in \mathbb {R}^n\Big \} \end{aligned} \end{aligned}$$

and

33 $$\begin{aligned} \mathcal {B}= \Big \{(0,t)\in \mathbb {R}^{n}\times \mathbb {R} \ : \ t<p^*\Big \} \end{aligned}$$

where $$p^*:=\inf _{x\in K}\left\Vert Ax-b\right\Vert _2^2$$.

### The Scalar Case

To clarify the general procedure, we focus on the simple case $m=n=1$ first, in which (28) becomes:

34 $$\begin{aligned} \text {minimize} \quad (Ax-b)^2, \qquad x\in \mathbb {R}, \ |x|\le \tau , \end{aligned}$$

where $A\in \mathbb {R}\setminus \{0\}$ and $b\in \mathbb {R}$. To find the Lagrange multipliers, we consider the set $\mathcal {G}$ of points $(u,t)\in \mathbb {R}^2$ that satisfy:

$$\begin{aligned} {\left\{ \begin{array}{ll} u= |x|-\tau ,\\ t= (Ax-b)^2, \end{array}\right. } \end{aligned}$$

which give a curve of the half-plane $U=\{(u,t)\in \mathbb {R}^2 \ : \ u\ge {-}\tau , \ t\ge 0\}$ parametrized by $x\in \mathbb {R}$. More precisely:

- if $$x\ge 0$$,
  $$\begin{aligned} {\left\{ \begin{array}{ll} x=u+\tau ,\\ t=\big (A(u+\tau )-b\big )^2=(Au+(A\tau -b))^2, \end{array}\right. } \end{aligned}$$
  which is a branch of parabola in *U* with vertex in $(\frac{b}{A}-\tau ,0)$.
- If $$x<0$$
  $$\begin{aligned} {\left\{ \begin{array}{ll} x=-u-\tau ,\\ t=\big (-A(u+\tau )-b\big )^2=(Au+(A\tau +b))^2, \end{array}\right. } \end{aligned}$$
  which is, again, a branch of parabola in *U*, having its vertex in $(-\frac{b}{A}-\tau ,0)$.

#### Proposition 3.1

Let $\tau >0$, $A\in \mathbb {R}{\setminus }\{0\}$, $b\in \mathbb {R}$. A Lagrange multiplier for (34) is given by:

$$\begin{aligned} \lambda ^\#={\left\{ \begin{array}{ll} 2A^2(|b/A|-\tau ) &{} \text {if}\, 0<\tau <|b/A|,\\ 0 &{} \text {if}\, \tau \ge |b/A| \end{array}\right. }=2A^2(|b/A|-\tau )^+. \end{aligned}$$

Namely, if $x^\#$ is a minimizer of (34), then it is also a minimizer for the problem:

$$\begin{aligned} \text {minimize} \quad (Ax-b)^2+\lambda ^\#|x|, \qquad x\in \mathbb {R}. \end{aligned}$$

### Properties of $\mathcal {A}$

Consider $A\in \mathbb {R}^{m\times n}$ and $b=(b_1,...,b_m)\in \mathbb {R}^m$, with:

$$\begin{aligned} A=\begin{pmatrix} a_{11} &{} \ldots &{} a_{1n}\\ \vdots &{} \ddots &{} \vdots \\ a_{m1} &{} \ldots &{} a_{mn} \end{pmatrix}. \end{aligned}$$

We consider the problem (28) and the associated Lagrange function:

35 $$\begin{aligned} L(x,\lambda ):=\left\Vert Ax-b\right\Vert _2^2+\sum _{j=1}^n\lambda _j(|x_j|-\tau _j). \end{aligned}$$

Recall that $p^*$ was defined as $p^*:=\min _{x\in K}\left\Vert Ax-b\right\Vert _2^2$, being *K* the set of the points $x\in {\mathbb {R}^n}$ such that $|x_j|\le \tau _j$ for all $j=1,\ldots ,n$. It is not difficult to verify that:

36 $$\begin{aligned} p^*=\inf \Big \{ t\in \mathbb {R}\ : \ (u,t)\in \mathcal {G}, \ u_j\le 0 \ \ \forall j=1,\ldots ,n\Big \}. \end{aligned}$$

Let $\mathcal {M}_n$ be the set of the *n*-dimensional signature matrices, that are the diagonal matrices $S=(s_{ij})_{i,j=1}^n\in \mathbb {R}^{n\times n}$ such that $|s_{jj}|=1$ for all $j=1,\ldots ,n$. Observe that if $S\in \mathcal {M}_n$, then $S^2=I_{n\times n}$, where $I_{n\times n}$ denotes the identity matrix in $\mathbb {R}^{n\times n}$, in particular *S* is invertible with $S^{-1}=S$. If $x\in \mathbb {R}^n$ and $S\in \mathcal {M}_n$ is such that $Sx\in \prod _{j=1}^n[0,+\infty )$, we write $S\in {{\,\textrm{sgn}\,}}(x)$.

#### Lemma 3.2

Let $A\in \mathbb {R}^{m\times n}$, $b\in \mathbb {R}^m$ and $\tau _j>0$ for $j=1,\ldots ,n$. Let $S\in \mathcal {M}_n$. There exists $u\in \prod _{j=1}^n[-\tau _j,0]$ such that $ASu+AS\tau -b=0$ if and only if $S\in {{\,\textrm{sgn}\,}}(x)$ for some $x\in \mathbb {R}^n$ such that $Ax=b$ and $|x_j|\le \tau _j$.

#### Proof

Assume that there exists $u\in \prod _{j=1}^n[-\tau _j,0]$ such that $ASu+AS\tau -b=0$ and let $x:=S(u+\tau )$. Then, $Sx=u+\tau \in \prod _{j=1}^n[0,\tau _j]$, so that $S\in {{\,\textrm{sgn}\,}}(x)$, $|x_j|\le \tau _j$ for all $j=1,\ldots ,n$ and

$$\begin{aligned} 0=AS(u+\tau )-b=Ax-b. \end{aligned}$$

Vice versa, assume that $Ax=b$ for some $x\in \prod _{j=1}^n[0,\tau _j]$. Let $S\in {{\,\textrm{sgn}\,}}(x)$ and $u:=Sx-\tau$. Then, $u\in \prod _{j=1}^n[-\tau _j,0]$ and

$$\begin{aligned} 0=Ax-b=A(Su+\tau )-b=ASu+AS\tau -b. \end{aligned}$$

$$\square $$

Recall the definitions of the two sets $\mathcal {A}$ and $\mathcal {B}$ given in (32) and (33) respectively. First, if $\mathcal {G}$ is the set of the points $(u,t)\in \mathbb {R}^{n+1}$ such that:

37 $$\begin{aligned} {\left\{ \begin{array}{ll} u_j= |x_j|-\tau _j &{} j=1,\ldots ,n,\\ t=\left\Vert Ax-b\right\Vert _2^2, \end{array}\right. } \end{aligned}$$

for some $x\in \mathbb {R}^n$, then

$$\begin{aligned} \mathcal {A}=\mathcal {G}+[0,+\infty )^{n+1}, \end{aligned}$$

that is, $(u,t)\in \mathcal {A}$ if and only if

38 $$\begin{aligned} {\left\{ \begin{array}{ll} u_j\ge |x_j|-\tau _j &{} j=1,\ldots ,n,\\ t\ge \left\Vert Ax-b\right\Vert _2^2, \end{array}\right. } \end{aligned}$$

for some $x\in \mathbb {R}^n$. Finally, $(u,t)\in \mathcal {B}$ if and only if $t<p^*=\min _{x_j\le \tau _j}\left\Vert Ax-b\right\Vert _2^2$.

We will prove that the equations (37) defining $\mathcal {G}$ can be written in terms of $\mathcal {M}_n$.

#### Lemma 3.3

Let $\tau _1,\ldots ,\tau _n>0$ and let $\mathcal {G}$ be the set of points satisfying (37). Then,

(i) $$\mathcal {G}$$ is closed.
(ii) $(u,p^*)\in \mathcal {G}$ for some $u\in \mathbb {R}^n$ such that $-\tau _j\le u_j\le 0$ for all $j=1,\ldots ,n$. Moreover, $p^*=\min \Big \{ t\in \mathbb {R}\ : \ (u,t)\in \mathcal {G}, \ u_j\le 0 \ \ \forall j=1,\ldots ,n\Big \}$.
(iii) For every $(u,t)\in \mathcal {G}$ there exists $S\in \mathcal {M}_n$ such that $t=\left\Vert ASu+(AS\tau -b)\right\Vert _2^2$. Viceversa, if $t=\left\Vert ASu+(AS\tau -b)\right\Vert _2^2$ for some $u\in \mathbb {R}^n$ such that $u_j\ge -\tau _j$ and some $S\in \mathcal {M}_n$, then $(u,t)\in \mathcal {G}$.

#### Proof

We prove that $\mathcal {G}$ is closed. For, let $(u^k,t^k)\in \mathcal {G}$ converge to $(u,t)\in \mathbb {R}^{n+1}$. We prove that $(u,t)\in \mathcal {G}$. Let $x^k\in \mathbb {R}^n$ be such that (37) is satisfied for $(u^k,t^k)$. Then, $|x^k_j|=u_j^k-\tau _j\le u_j+1-\tau _j$ for *j* sufficiently large. In particular, the sequence $\{x^k\}_k$ is bounded and, thus, it converges up to subsequences. Without loss of generality, we may assume that $(x^k)_k$ converges to $x:=\lim _{k\rightarrow +\infty }x^k$ in $\mathbb {R}^n$. Then, for all $j=1,\ldots ,n$,

$$\begin{aligned} |x_j|=\lim _{k\rightarrow +\infty }|x_j^k|=\lim _{k\rightarrow +\infty }u_j^k+\tau _j=u_j+\tau _j \end{aligned}$$

and, by continuity,

$$\begin{aligned} \left\Vert Ax-b\right\Vert _2^2=\lim _{k\rightarrow +\infty }\left\Vert Ax^k-b\right\Vert _2^2=\lim _{k\rightarrow +\infty }t^k=t. \end{aligned}$$

This proves that $(u,t)\in \mathcal {G}$ and, thus, that $\mathcal {G}$ is closed. (ii) follows by (i) and (36).

It remains to check (iii). If $(u,t)\in \mathcal {G}$, there exists $x\in \mathbb {R}^n$ satisfying (37). Let $S\in \mathcal {M}_n$ be such that $|x|=Sx$, where $|x|:=(|x_1|,\ldots ,|x_n|)$. Then, using the fact that $S^{-1}=S$,

$$\begin{aligned} |x|=u+\tau \ \ \ \Longrightarrow \ \ \ Sx=(u+\tau ) \ \ \ \Longrightarrow \ \ \ x=S(u+\tau ). \end{aligned}$$

By the last equation of (37), we have:

$$\begin{aligned} t=\left\Vert Ax-b\right\Vert _2^2=\left\Vert ASu+(AS\tau -b)\right\Vert _2^2. \end{aligned}$$

Viceversa, assume that $t=\left\Vert ASu+(AS\tau -b)\right\Vert _2^2$ for some $S\in \mathcal {M}_n$ and $u\in \mathbb {R}^n$ is such that $u\succeq -\tau$. Let $x:=S(u+\tau )$, then $|x_j|=|u_j+\tau _j|=u_j+\tau _j$ for all $j=1,\ldots ,n$ and $t=\left\Vert Ax-b\right\Vert _2^2$. This proves that $(u,t)\in \mathcal {G}$ and the proof of (iii) is concluded. $\square$

#### Lemma 3.4

Let $$u\in \prod _{j=1}^n[-\tau _j,+\infty )$$,

39 $$\begin{aligned} h_G(u):=\min _{S\in \mathcal {M}_n}\left\Vert ASu+AS\tau -b\right\Vert _2^2 \end{aligned}$$

and

$$\begin{aligned} g_G(u):=\min _{(u,s)\in \mathcal {G}}s. \end{aligned}$$

Then, $$h_G(u)=g_G(u)$$.

#### Proof

By Lemma 3.3 (iii), if $(u,s)\in \mathcal {G}$, then $s=\left\Vert AS_0u+AS_0\tau -b\right\Vert _2^2$ for some $S_0\in \mathcal {M}_n$. Hence,

$$\begin{aligned} h_G(u)=\min _{S\in \mathcal {M}_n}\left\Vert ASu+AS\tau -b\right\Vert _2^2\le \left\Vert AS_0u+AS_0\tau -b\right\Vert _2^2=s \end{aligned}$$

for all *s* such that $(u,s)\in \mathcal {G}$. Taking the minimum, we get $h_G(u)\le g_G(u)$. On the other hand, $(u,h_G(u))\in \mathcal {G}$ by Lemma 3.3 (iii). Therefore, $g_G(u)\le h_G(u)$ by definition of $g_G$. $\square$

#### Lemma 3.5

Let $\mathcal {G}$ be the set of points satisfying (37) and $\mathcal {A}$ be the set of points satisfying (38). Then,

(i) $$\mathcal {G}\subseteq \mathcal {A}$$;
(ii) $\mathcal {A}$ is closed.

#### Proof

(i) is obvious. We prove (ii).

Let $(u^k,t^k)\in \mathcal {A}$ be a sequence such that $(u^k,t^k)\xrightarrow [k\rightarrow +\infty ]{}(u,t)$ in $\mathbb {R}^{n+1}$. We need to prove that $(u,t)\in \mathcal {A}$. For all *k*, let $x^k\in \mathbb {R}^n$ be such that:

$$\begin{aligned} {\left\{ \begin{array}{ll} u^k_1\ge |x^k_1|-\tau _1,\\ \vdots \\ u_n^k\ge |x^k_n|-\tau _n,\\ t^k\ge \left\Vert Ax^k-b\right\Vert _2^2. \end{array}\right. } \end{aligned}$$

The sequence $\{x^k\}_k$ is bounded, in fact for all $j=1,\ldots ,n$, $|x_j^k|\le u_j^k+\tau _j\le u_j+1+\tau _j$ for *k* sufficiently large. Therefore, up to subsequences, we can assume $x^k\xrightarrow [k\rightarrow +\infty ]{}x$ in $\mathbb {R}^n$. For all $j=1,\ldots ,n$,

$$\begin{aligned} |x_j|=\lim _{k\rightarrow +\infty }|x_j^k|\le \lim _{k\rightarrow +\infty } u_j^k+\tau _j=u_j+\tau _j. \end{aligned}$$

Moreover, by continuity,

$$\begin{aligned} \left\Vert Ax-b\right\Vert _2^2&=\lim _{k\rightarrow +\infty }\left\Vert Ax^k-b\right\Vert _2^2\le \lim _{k\rightarrow +\infty }t^k=t. \end{aligned}$$

$$\square $$

#### Lemma 3.6

Let $\mathcal {A}$ be the set of points satisfying (38). Then,

(i) $\mathcal {A}$ is the epigraph of a convex non-negative function function $g:\prod _{j=1}^n[-\tau _j,+\infty )\rightarrow \mathbb {R}$ which is continuous in $\prod _{j=1}^n(-\tau _j,+\infty )$;
(ii) $$\partial g(0)\ne \varnothing $$;
(iii) $g(u)=0$ if and only if $(u,t)\in \mathcal {A}$ for all $t\ge 0$.

#### Proof

First, observe that $\mathcal {A}\subseteq \{(u,t) \, \ t\ge 0\}$ since $t\ge \left\Vert Ax-b\right\Vert _2^2\ge 0$ for some $x\in \mathbb {R}^n$ whenever $(u,t)\in \mathcal {A}$.

For the sake of completeness, we check that $\mathcal {A}$ is the epigraph of the function:

40 $$\begin{aligned} g(u)=\min _{(u,s)\in \mathcal {A}}s \ \ \ \ \left( u\in \prod _j[-\tau _j,+\infty )\right) , \end{aligned}$$

which is well defined by Lemma 3.5.

By the observation at the beginning of the proof, $g(u)\ge 0$. Let

$$\begin{aligned} epi(g):=\{(u,t) \, \ t\ge g(u)\} \end{aligned}$$

be the epigraph of *g*. If $(u,t)\in \mathcal {A}$, then $t\ge \min _{(u,s)\in \mathcal {A}}s=g(u)$, this means that $(u,t)\in epi(g)$. On the other hand, if $(u,t)\in epi(g)$, then $t\ge s$ for some $(u,s)\in \mathcal {A}$. But, if $t\ge s$ (and $(u,s)\in \mathcal {A}$), then $(u,t)\in \mathcal {A}$ as well, since $\mathcal {A}$ contains the vertical upper half-lines having their origins in (*u*, *s*), namely $(u,s)+(\{0\}\times [0,+\infty ))$.

This proves that $\mathcal {A}$ is an epigraph. Moreover, *g* is convex because $\mathcal {A}$ is convex (see [48] Proposition 2.4). The continuity of *g* on $\prod _j(-\tau _j,+\infty )$ follows from [47], Theorem 10.1. This proves (i).

Moreover, since $\tau _j>0$ for all $j=1,\ldots ,n$, $0\in \mathbb {R}^n$ is an interior point of $\prod _j[-\tau _j,+\infty )$. Since *g* is continuous and convex in $\prod _j(-\tau _j,+\infty )$, the subdifferential of *g* in 0 is non-empty and (ii) follows.

To prove (iii), assume that $g(u)=0$. Then, $\min _{(u,s)\in \mathcal {A}}s=0$ implies $(u,0)\in \mathcal {A}$. Since for all $t\ge 0$, $(u,0)+(\{0\}\times [0,+\infty ))\in \mathcal {A}$, we have that $(u,t)\in \mathcal {A}$ for all $t\ge 0$. For the converse, assume that $(u,t)\in \mathcal {A}$ for all $t\ge 0$. Then, $(u,0)\in \mathcal {A}$, so that (by the non-negativity of *g*) $0\le g(u)\le 0$. This proves the equivalence in (iii). $\square$

#### Remark 3.7

As we observed in the general theory situation, $(0,s)\in \mathcal {A}$ if and only if $s\ge p^*$. This tells that $g(0)=p^*$ and $(0,p^*)\in \mathcal {A}$.

We want to prove formally that *g*(*u*) defines the boundary $\partial \mathcal {A}$ of $\mathcal {A}$ in a neighborhood of $u=0$ and, then, find an explicit formula for *g*(*u*). Observe that, $\mathcal {A}=\partial \mathcal {A}\cup \mathring{\mathcal {A}}$, where $\mathring{\mathcal {A}}$ denotes the topologic interior of $\mathcal {A}$. Since $\mathcal {A}$ is closed and convex in $\mathbb {R}^n$, $\mathring{\mathcal {A}}$ coincides with the algebraic interior of $\mathcal {A}$, which is defined as follows:

#### Definition 3.8

Let *X* be a vector space and $\mathcal {A}\subseteq X$ be a subset. The **algebraic interior** of $\mathcal {A}$ is defined as:

$$\begin{aligned} {{\,\mathrm{a-int}\,}}(\mathcal {A}):=\{a\in \mathcal {A}\, \ \forall x\in X \ \exists \varepsilon _x>0 \ s.t. \ a+tx\in \mathcal {A}\ \forall t\in (-\varepsilon _x,\varepsilon _x) \}. \end{aligned}$$

#### Lemma 3.9

Let $\mathcal {A}$ be as in Lemma 3.5. Then,

41 $$\begin{aligned} \begin{aligned} \partial \mathcal {A}=&\{(u,t)\in \mathcal {A}\ : \ t=g(u), \ u_j>-\tau _j \ \forall j=1,\ldots , n\}\cup \\&\cup \{(u,t)\in \mathcal {A}\ : \ u_j=-\tau _j \ for \ some \ j=1,\ldots ,n\} \end{aligned} \end{aligned}$$

and the union is disjoint. Moreover,

$$\begin{aligned} \{(u,t)\in \mathcal {A}\, \ u_j= & {} -\tau _j \ for \ some \ j=1,\ldots ,n\}\\= & {} \{(u,t)\in \partial \mathcal {A}\, \ (u,t+\alpha )\in \partial \mathcal {A}\ \forall \alpha \ge 0\}. \end{aligned}$$

#### Proof

Observe that the union in (41) is clearly disjoint. We first prove (41).

($$\supseteq $$)
None of the sets on the RHS of (41) is contained in $\mathring{A}$. In fact,

- the definition of *g*(*u*) implies that for all $\varepsilon >0$, $-\varepsilon<t<\varepsilon$, $(u,t)\in \mathcal {A}$ if and only if $t\ge 0$, so that $(u,g(u))\notin {{\,\mathrm{a-int}\,}}(\mathcal {A})=\mathring{\mathcal {A}}$. This proves that the graph of *g* in $\prod _j(-\tau _j,+\infty )$ is a subset of $\partial \mathcal {A}$.
- Analogously, assume that $u_j=-\tau _j$ for some $j=1,\ldots ,n$, and for all $\varepsilon >0$ consider the point $(u_\varepsilon ,t)$, where $(u_\varepsilon )_l=u_l$ for all $l\ne j$ and $(u_\varepsilon )_j=-\tau _j-\varepsilon$. But *g* is defined on $\prod _j[-\tau _j,+\infty )$ and $\mathcal {A}$ is its epigraph, hence all the points of $\mathcal {A}$ must be in the form $(u,g(u)+\alpha )$ for some $u\in \prod _j[-\tau _j,+\infty )$, $t=g(u)+\alpha$ ($\alpha \ge 0$), hence $(u_\varepsilon ,t)\notin \mathcal {A}$ and this proves that $(u,t)\notin {{\,\mathrm{a-int}\,}}(\mathcal {A})$.

The fact that $\partial \mathcal {A}=\mathcal {A}\setminus \mathring{\mathcal {A}}$ proves the first inclusion.

($$\subseteq $$)
We prove that the complementary of the RHS of (41) in $\mathbb {R}^{n+1}$ is contained in $\mathring{A}$. Let (*u*, *t*) be such that $u>-\tau _j$ for all *j* and $t>g(u)$ (as it is easy to check, these are the conditions for (*u*, *t*) to belong to the complementary of the union of the two set at the LHS of (41)).

Let $d:=t-g(u)>0$. Since *g* is continuous on $\prod _j(-\tau _j,+\infty )$, there exists $\delta >0$ such that $|g(u)-g(v)|<d/4$ for all $v\in B_\delta (u):=\{w\in \mathbb {R}^n \ : \ |w-u|<\delta \}$. In particular, for all $v\in B_\delta (u)$, $g(v)<t-\frac{3}{4}d<t$. Then, $B_\delta (u)\times (t-\frac{3}{4}d,+\infty )$ is all contained in $\mathcal {A}$ (because $\mathcal {A}$ is the epigraph of *g*) and it is an open neighborhood of (*u*, *t*). Hence, $(u,t)\in \mathring{A}=\mathcal {A}\setminus \partial \mathcal {A}$.

Next, we check the second part of the lemma:

($$\subseteq $$)
assume $(u,t)\in \mathcal {A}$ is such that $u_j=-\tau _j$ for some *j*. Then, by the first part of this Lemma, $(u,t+\alpha )\in \partial \mathcal {A}$ for all $\alpha \ge 0$, since (41) is a partition of $\partial \mathcal {A}$.
($$\supseteq $$)
Assume that $(u,t+\alpha )\in \partial \mathcal {A}$ for all $\alpha \ge 0$. Then, $(u,t)\in \partial \mathcal {A}$. Assume by contradiction that $u_j>-\tau _j$ for all *j*. Then, since (41) is a partition of $\partial \mathcal {A}$, $g(u)=t+\alpha$ for all $\alpha \ge 0$, which cannot be the case.

$$\square $$

The function *g*, defined in Lemma 3.6, can be expressed in terms of the function $h_G$ of Lemma 3.4, as shown in the following result.

#### Theorem 3.10

Let $\mathcal {A}$ be the set of points satisfying (38), $h_G$ and *g* be the functions defined in (39) and (40), respectively. For $u\in \prod _{j=1}^n[-\tau _j,+\infty )$, $u=(u_1,\ldots ,u_n)$, let $Q(u):=\prod _{j=1}^n[-\tau _j,u_j]$ and

42 $$\begin{aligned} h(u):=\min _{S\in \mathcal {M}_n, \ v\in Q(u)}\left\Vert AS(v+\tau )-b\right\Vert _2^2=\min _{v\in Q(u)}h_G(v). \end{aligned}$$

Then, $h(u)=g(u)$ for all $u\in \prod _j[-\tau _j,+\infty )$.

#### Proof

We first prove that $g(u)\le h(u)$. For, it is enough to prove that $(u,h(u))\in \mathcal {A}$, so that $g(u)\le h(u)$ would follow by the definition of *g*. By definition of *h*, there exist $S_0\in \mathcal {M}_n$ and $v\in Q(u)$ so that:

$$\begin{aligned} h(u)=\left\Vert AS_0v+AS_0\tau -b\right\Vert _2^2. \end{aligned}$$

By Lemma 3.3 (iii), $(v,h(u))\in \mathcal {G}$. Since $u_j\ge v_j$ for all $j=1,\ldots ,n$, it follows that $(u,h(u))\in \mathcal {A}$ by definition of $\mathcal {A}$.

For the converse, since $(u,g(u))\in \mathcal {A}$, there exists $(v',t)\in \mathcal {G}$ such that $v_j'\le u_j$ for all $j=1,\ldots ,n$ and $g(u)\ge t$. In particular, $v'\in Q(u)$. By Lemma 3.3 (iii), $t=\left\Vert AS_1v'+AS\tau -b\right\Vert _2^2$ for some $S_1\in \mathcal {M}_n$. Therefore,

$$\begin{aligned} g(u)\ge \left\Vert AS_1v'+AS_1\tau -b\right\Vert _2^2\ge \min _{S\in \mathcal {M}_n, \ v\in Q(u)}\left\Vert ASv+AS\tau -b\right\Vert _2^2= h(u). \end{aligned}$$

This concludes the proof. $\square$

Even if $g=h$, in what follows we still distinguish *h* and *g* when we want to stress the explicit definitions of both. Namely, we write *g*(*u*) when we refer to $\min _{(u,s)\in \mathcal {A}}s$ and *h*(*u*) when we refer to (42).

#### Corollary 3.11

Under the same notation as above,

43 $$\begin{aligned} g(u)=\min _{-u-\tau \preceq v\preceq u+\tau }\left\Vert Av-b\right\Vert _2^2. \end{aligned}$$

#### Proof

Using the second expression in (42),

$$\begin{aligned} g(u)=\min _{S\in \mathcal {M}_n}\min _{v\in Q(u)}\left\Vert AS(v+\tau )-b\right\Vert _2^2. \end{aligned}$$

But,

$$\begin{aligned} f_S(v)=\left\Vert AS(v+\tau )-b\right\Vert _2^2=f(S(v+\tau )), \end{aligned}$$

for $f(v)=\left\Vert Av-b\right\Vert _2^2$, that gives:

$$\begin{aligned} \min _{-\tau \preceq v\preceq u}f_S(v)=\min _{v\in Q(u)}f(S(v+\tau ))=\min _{v\in S(Q(u)+\tau )}\left\Vert Av-b\right\Vert _2^2, \end{aligned}$$

so that:

$$\begin{aligned} \min _{S\in \mathcal {M}_n}\min _{-\tau \preceq v\preceq u}f_S(v)=\min _{\bigcup _{S\in \mathcal {M}_n}S(Q(u)+\tau )}\left\Vert Av-b\right\Vert _2^2 \end{aligned}$$

and the assertion follows by observing that

$$\begin{aligned} \bigcup _{S\in \mathcal {M}_n}S(Q(u)+\tau )=\{v\in \mathbb {R}^n: -u-\tau \preceq v\preceq u+\tau \}. \end{aligned}$$

$$\square $$

### A Result Under Conditions on the Gradient of $\left\Vert Ax-b\right\Vert _2^2$

In general, the geometry of $\mathcal {A}$ is so complicated that expressing *g* explicitly may turn into a tough task. Nevertheless, it is obvious that if *u* is itself one of the minimizers of (42), then $g(u)=h_G(u)=\min _{S\in \mathcal {M}_n}\left\Vert ASu+AS\tau -b\right\Vert _2^2$. So, under further assumptions on $\nabla (\left\Vert ASu-b\right\Vert _2^2)$ granting the equality $g(u)=h_G(u)$ holds in a neighborhood of 0, we can compute explicitly the Lagrange multipliers.

#### Theorem 3.12

Let $f(v)=\left\Vert Av-b\right\Vert _2^2$ and assume that for all $k=1,\ldots ,n$ the condition:

44 $$\begin{aligned} \sum _{j=1}^nu_j\langle a_{*,j},a_{*,k}\rangle \le \langle b,a_{*,k}\rangle \qquad (-\tau \preceq u\preceq \tau ) \end{aligned}$$

holds. Then, $g(u)=f(u+\tau )$ for all $u\in Q(0)$ and $\lambda ^\#=A^T(b-A\tau )$ is a set of Lagrange multipliers for problem (28).

#### Proof

The set of conditions (44) is equivalent to $(Au-b)^TA\preceq 0$ for all $-\tau \preceq u\preceq \tau$, that is $\nabla f(u)\preceq 0$ for $-\tau \preceq u\preceq \tau$. We prove that, under this further condition, $g(u)=f(u+\tau )$ for all $u\in Q(0)$. Let $u\in Q(0)$ and $\mathfrak {n}\succ 0$ be a unit vector. For all $t\in \mathbb {R}$, define:

$$\begin{aligned}\begin{aligned} f_{\mathfrak {n}}(t)&:=f(u+\tau +t\mathfrak {n})=\left\Vert A(u+\tau +t\mathfrak {n})-b\right\Vert _2^2\\&=\left\Vert A\mathfrak {n}\right\Vert _2^2t^2+2\langle A(u+\tau )-b, A\mathfrak {n} \rangle t +\left\Vert A(u+\tau )-b\right\Vert _2^2, \end{aligned}\end{aligned}$$

which is the restriction of *f* to the line $\{u+t\mathfrak {n} : t\in \mathbb {R}\}$. If $\mathfrak {n}\in \text {ker}(A)$, then $f_{\mathfrak {n}}\equiv 0$ and it has a global minimum in $t=0$. Assume $\mathfrak {n}\notin \text {ker}(A)$. The intersection of this line with $\{-\tau \preceq v\preceq \tau \}$ is contained in $(-\infty ,0]$. If we prove that, for all $\mathfrak {n}\succ 0$, $f_{\mathfrak {n}}$ has a constrained minimum in $t=0$, we get the first assertion. For, it’s enough to observe that

$$\begin{aligned} f'_{\mathfrak {n}}(0)=\nabla f(u+\tau )\cdot \mathfrak {n}\le 0, \end{aligned}$$

because if $u\in Q(0)$, then $\{-u-\tau \preceq v \preceq u+\tau \}\subseteq \{-\tau \preceq v\preceq \tau \}$. This proves that $g(u)=f(u+\tau )$ for all $u\in Q(0)$. In particular,

$$\begin{aligned} -\nabla g(0)=-\nabla f(\tau )=(b-A\tau )^TA\succeq 0 \end{aligned}$$

is a set of Lagrange multipliers for (28). $\square$

#### Remark 3.13

It is not difficult to generalize Theorem 3.12 a bit further. If the hyperparallelogram $\{-\tau \preceq u\preceq \tau \}$ is all contained in the region $\{u\in \mathbb {R}^n \ : \ S\nabla f(u)\preceq 0\}$ for some $S\in \mathcal {M}_n$, then $g(u)=f(S(u+\tau ))$ for all $u\in Q(0)$ and

$$\begin{aligned} \lambda ^\#=-\nabla g(0)=-S\nabla f(S(u+\tau ))^T \end{aligned}$$

defines a vector of Lagrange multipliers for (28). The proof goes exactly as in Theorem 3.12.

### Decoupling the Variables

In this subsection, we focus on the situation in which $A^TA$ is a diagonal matrix. Since:

$$\begin{aligned} A^TA=\begin{pmatrix} \left\Vert a_{*,1}\right\Vert _2^2 &{} \langle a_{*,1},a_{*,2}\rangle &{} \ldots &{} \langle a_{*, 1},a_{*,n}\rangle \\ \langle a_{*,2},a_{*,1}\rangle &{} \left\Vert a_{*, 2}\right\Vert _2^2 &{} \ldots &{} \langle a_{*, 2},a_{*, n}\rangle \\ \vdots &{} \vdots &{} \ddots &{} \vdots \\ \langle a_{*,n},a_{*,1}\rangle &{} \langle a_{*,n},a_{*, 2}\rangle &{} \ldots &{} \left\Vert a_{*, n}\right\Vert _2^2 \end{pmatrix} \end{aligned}$$

and the rank of $A^TA$ is equal to that of *A*, it follows that in this case:

45 $$\begin{aligned} A^TA={{\,\textrm{diag}\,}}(\left\Vert a_{*,1}\right\Vert _2^2,...,\left\Vert a_{*, n}\right\Vert _2^2). \end{aligned}$$

#### Remark 3.14

If $m\le n$ and $A^TA$ is diagonal, $n-m$ of the norms in (45) above vanish. In this case, we assume that $a_{*,m+1}=...=a_{*, n}=0$, so that *A* can be written in terms of its columns as:

$$\begin{aligned} A=\begin{pmatrix}A' | 0_{m\times (n-m)}\end{pmatrix}, \end{aligned}$$

where $A'=(a_{*, 1}|...|a_{*, m})\in GL(m,\mathbb {R})$. Observe that:

$$\begin{aligned} \left\Vert Ax-b\right\Vert _2^2=\left\Vert A'x'-b\right\Vert _2^2, \end{aligned}$$

where $x'=(x_1,...,x_m)^T$, so that $x^\#$ is a mimizer of (28) if and only if $(x^\#)'=(x^\#_1,\ldots ,x^\#_m)$ is a minimizer of the problem:

46 $$\begin{aligned} \text {minimize}\quad \left\Vert A'y-b\right\Vert _2^2, y\in \mathbb {R}^m, \ |y_j|\le \tau _j,\ j=1,\ldots ,m, \end{aligned}$$

under the further condition that the remaining coordinates of *x* vanish.

For this reason, for the rest of this subsection, we focus on (46), both for the cases $n\le m$ and $m\le n$, and provide the Lagrange multipliers.

#### Remark 3.15

We point out that in this situation the Lagrange multipliers can be computed directly from Proposition 3.1. Indeed, under the *orthogonality* assumption on *A*, the target function in problem (46) becomes:

$$\begin{aligned} \sum _{j=1}^m(\left\Vert a_{*,j}\right\Vert ^2_2y_j^2-2y\langle a_{*,j},n\rangle y_j)+\left\Vert b\right\Vert _2^2. \end{aligned}$$

Since the variables of all the addenda are decoupled, and the addenda are non-negative,

$$\begin{aligned}{} & {} \min _y\sum _{j=1}^m(\left\Vert a_{*,j}\right\Vert ^2_2y_j^2-2y\langle a_{*,j},n\rangle y_j)+\left\Vert b\right\Vert _2^2\\{} & {} \quad =\sum _{j=1}^m\min _{y_j}\left( \left\Vert a_{*,j}\right\Vert ^2_2y_j^2-2y\langle a_{*,j},n\rangle y_j+\frac{\left\Vert b\right\Vert _2^2}{m}\right) \end{aligned}$$

and a minimizer of (46) is also a minimizer of the problem:

$$\begin{aligned} \text {minimize} \quad \left\Vert a_{*,j}\right\Vert ^2_2y_j^2-2y\langle a_{*,j},n\rangle y_j+\frac{\left\Vert b\right\Vert _2^2}{m},\qquad |y_j|\le \tau _j \end{aligned}$$

for all $j=1,...,m$. In other words, it is enough to treat (46) as *m* 1-dimensional constrained minimization problems. However, our interest is testing the tools presented in the previous section, computing the function *g* and the separating hyperplane.

To exhibit a vector of Lagrange multipliers, we start by the set

$$\begin{aligned} \mathcal {G}:= & {} \{(u,t)\in \mathbb {R}^{m+1} \, \ u_j=|y_j|-\tau _j \ (j=1,\ldots ,m), \\{} & {} t=\left\Vert A'y-b\right\Vert _2^2 \ \text {for some}\, y\in \mathbb {R}^m\}. \end{aligned}$$

By Lemma 3.3 (iii), $(u,t)\in \mathcal {G}$ if and only if $u\succeq -\tau$ and $t=\left\Vert A'S(u+\tau )-b\right\Vert _2^2$ for some $S\in \mathcal {M}_m$. Let $f_S(u)=\left\Vert A'S(u+\tau )-b\right\Vert _2^2$ and observe that:

$$\begin{aligned} f_S(u)=\sum _{j=1}^m\left\Vert a_{*,j}\right\Vert _2^2(u_j+\tau _j)^2-2\sum _{j=1}^ms_{jj}\langle b,a_{*,j} \rangle (u_j+\tau _j)+\left\Vert b\right\Vert _2^2. \end{aligned}$$

The functions $f_S$ are the equivalent of the parabolas in the 1-dimensional case and they describe elliptic paraboloids. As it clear by Sect. 3.1, we need to understand what is $h_G(u):=\min _{S\in \mathcal {M}_m}f_S(u)$. Observe that for all $S\in \mathcal {M}_n$,

47 $$\begin{aligned} f_S(u)\ge \sum _{j=1}^m\left\Vert a_{*, j}\right\Vert _2^2(u_j+\tau _j)^2-2\sum _{j=1}^m|\langle b,a_{*,j} \rangle |(u_j+\tau _j)+\left\Vert b\right\Vert _2^2=f_{S_\beta }(u), \end{aligned}$$

where $S_\beta =(s_j^\beta )_{j=1}^m\in \mathcal {M}_m$ is a diagonal matrix such that $s_j^\beta \langle b,a_{*,j}\rangle \ge 0$.

#### Lemma 3.16

Under the notation and the assumptions of this subsection,

$$\begin{aligned} h_G(u)=f_{S_\beta }(u)=\sum _{j=1}^m\left\Vert a_{*,j}\right\Vert _2^2(u_j+\tau _j)^2-2\sum _{j=1}^m|\langle b,a_{*, j} \rangle |(u_j+\tau _j)+\left\Vert b\right\Vert _2^2. \end{aligned}$$

$h_G$ defines an elliptic paraboloid whose vertex $V=(c,0)\in \mathbb {R}^{m+1}$ is characterized both by $c=-\tau +S_\beta (A')^{-1}b$ and

$$\begin{aligned} c_j=-\tau _j+\frac{|\langle b,a_{*,j}\rangle |}{\left\Vert a_{*,j}\right\Vert _2^2} \end{aligned}$$

($$j=1,\ldots ,m$$). Moreover,

48 $$\begin{aligned} h_G(u)=\sum _{j=1}^m\left\Vert a_{*,j}\right\Vert _2^2(u_j-c_j)^2. \end{aligned}$$

#### Proof

We already proved the first part of the Lemma. We only need to compute the vertex of $f_{S_\beta }$. For, observe that the minimum of $f_{S_\beta }$ is (*c*, 0), where *c* satisfies $f_S(c)=0$. This equation is satisfied if and only if $c=-\tau +S_\beta (A')^{-1}b$. Moreover, the minimum of $f_{S_\beta }$ is also characterized by $\nabla f_{S_\beta }(c)=0$, that is:

$$\begin{aligned} c_j+\tau _j-\frac{|\langle b,a_{*,j}\rangle |}{\left\Vert a_{*,j}\right\Vert _2^2}=0 \end{aligned}$$

($j=1,\ldots ,m$). Finally, using the first characterization of *c*,

$$\begin{aligned} h_G(u)&=\left\Vert AS_\beta (u+\tau )-b\right\Vert _2^2=\left\Vert AS_\beta (u-c)+AS_\beta (c+\tau )-b\right\Vert _2^2\\&=\left\Vert AS_\beta (u-c)\right\Vert _2^2=\\&=\sum _{j=1}^m\left\Vert a_{*,j}\right\Vert _2^2(u_j-c_j)^2. \end{aligned}$$

This concludes the proof. $\square$

In order to compute the Lagrange multipliers for the decoupled problem, we observe that $\mathcal {A}+[0,+\infty )^{m+1}$ is the epigraph of the function *g*(*u*) whose first properties are proved in Lemma 3.6. Hence, this function describes the lower boundary of $\mathcal {A}$, that is the part of $\mathcal {A}$ we need to compute a separating hyperplane. By (42), $g(u)=\min _{v\in Q(u)}h_G(v)$, where $Q(u)=\prod _{j=1}^m[-\tau _j,u_j]$.

#### Theorem 3.17

Under the notation and the assumptions of this subsection,

$$\begin{aligned} g(u)=h_G(Pu), \end{aligned}$$

where $P:\prod _{j=1}^m[-\tau _j,+\infty )\rightarrow Q(c)$ is the projection defined for all $u\in \prod _{j=1}^m[-\tau _j,+\infty )$ by

49 $$\begin{aligned} (Pu)_j={\left\{ \begin{array}{ll} u_j &{} \text {if}\, -\tau _j\le u_j\le c_j,\\ c_j &{} \text {if}\,u_j>c_j \end{array}\right. }=\min \{c_j,u_j\} \end{aligned}$$

($j=1,\ldots ,m$). Explicitly, under the assumptions of this subsection,

50 $$\begin{aligned} g(u)=\sum _{j=1}^m\left\Vert a_{*,j}\right\Vert _2^2(u_j-c_j)^2\chi _{[-\tau _j,c_j]}(u_j). \end{aligned}$$

In particular, $g\in \mathcal {C}^1(\prod _{j=1}^n(-\tau _j,+\infty ))$ with:

51 $$\begin{aligned} \frac{\partial g}{\partial u_j}(u)= 2\left\Vert a_{*,j}\right\Vert _2^2(u_j-c_j)\chi _{[-\tau _j,c_j]}(u_j) \end{aligned}$$

for all $$u\in \prod _{j=1}^n(-\tau _j,+\infty )$$.

#### Proof

Obviously, *P* is a projection of $\prod _{j=1}^n[-\tau _j,+\infty )$ onto *Q*(*c*). For all $j=1,\ldots ,m$,

$$\begin{aligned} \text {argmin}_{-\tau _j\le v_j\le u_j}\left\Vert a_{*,j}\right\Vert _2^2(v_j-c_j)^2={\left\{ \begin{array}{ll} u_j &{} \text {if}\, -\tau _j\le u_j\le c_j,\\ c_j &{} \text {otherwise} \end{array}\right. }=(Pu)_j. \end{aligned}$$

Hence,

$$\begin{aligned}\begin{aligned} g(u)&=\min _{v\in Q(u)}h_G(v)=\sum _{j=1}^m\min _{-\tau _j\le v_j\le u_j}\left\Vert a_{*,j}\right\Vert _2^2(v_j-c_j)^2\\&=\sum _{j=1}^m\left\Vert a_{*,j}\right\Vert _2^2((Pu)_j-c_j)^2=h_G(Pu). \end{aligned} \end{aligned}$$

The explicit definition of *Pu* gives (50) and (51). The differentiability and formula (51) are obvious by the expression (50) of *g*. $\square$

#### Remark 3.18

As a consequence of Theorem 3.17,

$$\begin{aligned} p^*=g(0)=\sum _{j=1}^m\left\Vert a_{*,j}\right\Vert _2^2\left( -\tau _j+\frac{|\langle b,a_{*,j}\rangle |}{\left\Vert a_{*,j}\right\Vert _2^2}\right) ^2\chi _{[-\tau _j,c_j]}(0). \end{aligned}$$

Then, observe that:

52 $$\begin{aligned} -\tau _j\le 0\le -\tau _j+\frac{|\langle b,a_{*,j}\rangle |}{\left\Vert a_{*,j}\right\Vert _2^2} \ \ \ \Longleftrightarrow \ \ \ 0\le \tau _j\le \frac{|\langle b,a_{*,j}\rangle |}{\left\Vert a_{*,j}\right\Vert _2^2}, \end{aligned}$$

so that:

$$\begin{aligned} p^*=\sum _{j=1}^m\left\Vert a_{*,j}\right\Vert _2^2\left( -\tau _j+\frac{|\langle b,a_{*,j}\rangle |}{\left\Vert a_{*,j}\right\Vert _2^2}\right) ^2\chi _{\Big [0,\frac{|\langle b,a_{*,j}\rangle |}{\left\Vert a_{*,j}\right\Vert _2^2}\Big ]}(\tau _j). \end{aligned}$$

#### Theorem 3.19

Under the notation of this subsection, the vector $\lambda ^\#\in [0,+\infty )^m$ given by

$$\begin{aligned} \lambda _j^\#=2\left\Vert a_{*,j}\right\Vert _2^2\left( \frac{|\langle b,a_{*,j}\rangle |}{\left\Vert a_{*,j}\right\Vert _2^2}-\tau _j\right) ^+ \end{aligned}$$

defines a vector of Lagrange multipliers for (46).

#### Proof

We apply (51) to $u=0$ and use (52). Namely,

$$\begin{aligned} t=p^*+\langle \nabla g(0),u\rangle \end{aligned}$$

is the tangent hyperplane of *g* in $u=0$, which is also the hyperplane that separates $\mathcal {A}$ and $\mathcal {B}$. The direction of this hyperplane is $(\nabla g(0),-1)$, so that:

$$\begin{aligned} \lambda ^\#=-\nabla g(0), \end{aligned}$$

i.e. the assertion. $$\square $$

#### Remark 3.20

As far as the original problem (28) with $m\le n$ is concerned, we get the Lagrange multipliers for free by Theorem 3.19 simply observing that if $A=(a_{*1}|\ldots |a_{*m}|0|\ldots |0)\in \mathbb {R}^{m\times n}$, $A'=(a_{*1}|\ldots |a_{*m})$ and $x=(x',x'')\in \mathbb {R}^m\times \mathbb {R}^{n-m}$, then

$$\begin{aligned}\begin{aligned} \min _{x\in \mathbb {R}^n}\left\Vert Ax-b\right\Vert _2^2+\sum _{j=1}^n\lambda _j(|x_j|-\tau _j)=&\min _{x'\in \mathbb {R}^m}\left\Vert A'x'-b\right\Vert _2^2+\sum _{j=1}^m\lambda _j(|x'_j|-\tau _j)+\\&+\underbrace{\min _{x''\in \mathbb {R}^{n-m}}\sum _{j=m+1}^n\lambda _j(|x_j|-\tau _j)}_=-\sum _{j=m+1}^n\lambda _j\tau _j, \end{aligned} \end{aligned}$$

so that, if $\lambda ^\#\in \mathbb {R}^m$ defines a vector of Lagrange multipliers for (46), then $(\lambda ^\#|0)\in \mathbb {R}^m\times \mathbb {R}^{n-m}$ defines a vector of Lagrange multipliers for (28).

### Explicit Solution

The conditions $|x_j|\le \tau _j$ are equivalent to $x_j^2\le \tau _j^2$. Under this point of view, (28) can be restated as:

53 $$\begin{aligned} \text {minimize} \quad \left\Vert Ax-b\right\Vert _2^2,\qquad x_j^2\le \tau _j^2, \end{aligned}$$

that can be interpreted as a weighted Tikhonov problem. Assume that $\lambda ^\#$ is a vector of Lagrange multipliers for (28) or, equivalently, for (53). We are interested in computing

$$\begin{aligned} x^\#=\arg \min _xL(x,\lambda ), \end{aligned}$$

where *L* is the Lagrange function associated to (53), i.e.

$$\begin{aligned} L(x,\lambda ^\#)=\left\Vert Ax-b\right\Vert _2^2+\sum _{j=1}^n\lambda _j^\#(x_j^2-\tau _j^2). \end{aligned}$$

Since $L\in \mathcal {C}^\infty (\mathbb {R}^n)$ and it is convex, they satisfy $\nabla L(x,\lambda ^\#)=0$, that is:

$$\begin{aligned} (A^TA+\Delta _\lambda )x=A^Tb, \end{aligned}$$

where $\Delta _\lambda ={{\,\textrm{diag}\,}}(\lambda _1^\#,...,\lambda _n^\#)$. Hence, $x^\#$ satisfies:

54 $$\begin{aligned} (A^TA+\Delta _\lambda )x^\#=A^Tb, \end{aligned}$$

that is, $$x^\#\in (A^TA+\Delta _\lambda )^{-1}A^Tb$$.

#### Remark 3.21

Another way to compute the Lagrange multipliers associated to (28), or equivalently to (53), can be by means of strong duality condition, namely using:

$$\begin{aligned} \lambda ^\#=\arg \max _{\lambda \succeq 0}\min _x L(x,\lambda ). \end{aligned}$$

However, we stress that the explicit value of $\min _x L(x,\lambda )$ is still hard to compute since the implicit relation (54) satisfied by $x^\#$ cannot be made explicit by means of Dini’s theorem.

## Considerations and Conclusions

### Applications

Despite the apparently heavy assumptions on *A*, Theorem 3.19 has itself interesting applications. For instance, it can be applied to denoising problems, where $A=I_{n\times n}$, i.e. problems in the form:

55 $$\begin{aligned} \text {minimize}\quad \left\Vert x-b\right\Vert _2^2, \qquad x\in \mathbb {R}^n, \ |x_j|\le \tau _j, \ j=1,\ldots ,n. \end{aligned}$$

By Theorem 3.19, $\lambda ^\#=(\lambda ^\#_j)_{j=1}^n$ is a vector of Lagrange multipliers for (55), where:

56 $$\begin{aligned} \lambda ^\#_j=2(|b_j|-\tau _j)^+. \end{aligned}$$

We can also apply Theorem 3.19 to the discrete Fourier transform, i.e. given a noisy fully-sampled signal $b\in \mathbb {C}^{n}$, we want to find a vector $z\in \mathbb {C}^{n}$ such that $\left\Vert \Phi z-b\right\Vert _2^2$ is minimized under the constrains $|z_j|\le \tau _{j}$, where $\Phi \in \mathbb {C}^{n \times n}$ denotes the (complex) DFT matrix. Since $\Phi ^*\Phi =I_{n\times n}$, we can apply Theorem 3.19 to deduce that a set of Lagrange multipliers for this problem is:

$$\begin{aligned} \lambda _j^\#=2\left( |\langle b,\phi _{*,j}\rangle |-\tau _j\right) ^+, \end{aligned}$$

($j=1,\ldots ,n$), being $\phi _{*,j}$ the *j*-th column of $\Phi$.

The question that naturally arises in the applications is whether the dependence of $\lambda _1,\ldots ,\lambda _n$ on $\tau _1,\ldots ,\tau _n$ can be a critical issue in the applicability of the theory. Indeed, $\tau _1,\ldots ,\tau _n$ are upper bounds for $|x_1|,\ldots ,|x_n|$ respectively, which are not available in the practice. However, whenever it is possible to estimate these local upper bounds, our result may lead to high-quality imaging perfomances. For instance, for denoising, (56) may be approximated by replacing $\tau _1,\ldots ,\tau _n$ with the voxel values obtained by applying a Gaussian filter (or other types of filtering) to the noisy image. This opens the question of which filtering technique could lead to optimal approximations of the $\tau _1,\ldots ,\tau _n$ depending on the field of research in which (28) can be implemented. We intend to investigate this topic in the immediate future.

### Open Problems

As long as $A^TA$ is not a diagonal matrix, the geometries of the sets $\mathcal {G}$ and $\mathcal {A}$ of the points satisfying (37) and (38) respectively, become more involved, along with the possible casuistry. However, the general case in which $A^TA$ is not diagonal would be of great importance in applications. Indeed, as long as Lagrange multipliers are proved to act as effective tuning parameters, the behavior of Lagrange multipliers for the weighted LASSO problem (13) in terms of voxel-wise estimates would provide a way to control the tuning parameters via estimates of the $\tau _j$.

Another open problem is whether it is possible to apply the same procedure to compute the Lagrange multipliers for (3). Clearly, the corresponding sets $\mathcal {G}$ and $\mathcal {A}$ lie in $\mathbb {R}^2$ so that $g:\mathbb {R}\rightarrow \mathbb {R}$. Despite this simplifying fact, the set $\mathcal {G}$ is characterized by:

$$\begin{aligned} {\left\{ \begin{array}{ll} u = s(x)^Tx-\tau ,\\ t = \left\Vert Ax-b\right\Vert _2^2 \end{array}\right. } \qquad \text {for some}\, x\in \mathbb {R}^n, \end{aligned}$$

where $s(x)\in \mathbb {R}^n$ is a vector such that ${{\,\textrm{diag}\,}}(s(x)_j) \in {{\,\textrm{sgn}\,}}(x)$ and, in this case, *u* and *x* belong to different spaces and a closed form for $t=t(u)$ is even more difficult to provide.

The possibility of using Lagrange multipliers as tuning parameters in disciplines that apply LASSO problems, such as MRI, is still open. Lagrange multipliers for (6), however, depend on its constraint:

$$\begin{aligned} \left\Vert Ax-b\right\Vert _2\le \eta . \end{aligned}$$

Consequently, even if the utilization of Lagrange multipliers as tuning parameters were feasible in applications, unless a method for accurately estimating $\eta$ is provided, the focus would simply shift from Lagrange multipliers to estimating the $\ell _2$ norm of the noise. It is therefore crucial to determine whether slight perturbations in these estimates lead to significant variations, for instance, of the quality of retrieved images in MRI.

Finally, we stress that it would be important to generalize (28) up to consider different inner products on $\mathbb {R}^n$. Namely, this is the situation that occurs in MRI when the undersampling pattern is non-cartesian. Problem (13) in this case becomes:

$$\begin{aligned} \min _x\left\Vert Ax-b\right\Vert _W^2+\sum _j\lambda _j|x_j|, \end{aligned}$$

where

$$\begin{aligned} \left\Vert x\right\Vert _W=x^TW^TWx \qquad (x\in \mathbb {R}^n), \end{aligned}$$

for a definite positive diagonal matrix *W*. Since this topic falls beyond the purpose of this work, we limit ourselves to mention the very mathematical reason why the weighted norm shall definitely replace the Euclidean norm over $\mathbb {R}^n$ when sampling is not performed on a cartesian grid. Indeed, non-cartesian sampling patterns require appropriate discretizations of the Fourier transform integral. Roughly speaking,

$$\begin{aligned} {\hat{f}}(\xi )\thickapprox \sum _j f(x_j)e^{-2\pi i\xi \cdot x_j}\Delta x_j=\langle f,e^{2\pi i\xi \cdot }\rangle _W, \end{aligned}$$

where $\Delta x_j$ is the Lebesgue measure of an adequate neighborhood of $x_j$, weighting the contribution of the sample $x_j$, and *W* is the diagonal matrix whose entries are $\sqrt{\Delta x_j}$. The inversion formula of the Fourier transform shall be modified accordingly. For instance, if the sampling follows a spiral trajectory, $\Delta x_j$ shall be bigger the further $x_j$ is from the origin, since this value serves as an avarage of *f* on a portion of sphere that is larger as $x_j$ is far from the origin. All the above-mentioned problems will be object of our future investigations.

## Appendix

Since we did not find a direct proof in the existing literature, we provide a formal proof of the existence of the minimizer of the generalized LASSO problem:

57 $$\begin{aligned} \arg \min _{x\in \mathbb {R}^n}\left\Vert Ax-b\right\Vert _2^2+\lambda \left\Vert \Phi x\right\Vert _1, \end{aligned}$$

where $$b\in \mathbb {R}^m$$, $$A\in \mathbb {R}^{m\times n}$$ and $$\Phi \in \mathbb {R}^{N\times n}$$.

We state the result in the general framework of finite-dimensional vector spaces: we denote by *X*, *Y*, *Z* three finite-dimension real vector spaces. We denote by $\langle \cdot ,\cdot \rangle _X$ an inner product on *X* and with $\left\Vert \cdot \right\Vert _X$ the induced norm. Analogous notation will be used for *Y*, whereas we set:

$$\begin{aligned} \left\Vert z\right\Vert _p=\left( \sum _{j=1}^{\text {dim}(Z)}|z_j|^p\right) ^{1/p},\qquad z\in Z \end{aligned}$$

for $0<p\le \infty$. Recall that $\left\Vert \cdot \right\Vert _p$ is a Banach quasi-norm (meaning that there exists $C_p\ge 1$ such that $\left\Vert x+y\right\Vert _p\le C_p(\left\Vert x\right\Vert _p+\left\Vert y\right\Vert _p)$ for all $x,y\in Z$) replaces the triangular inequality for $0<p<1$, and it is a norm for $1\le p\le \infty$.

Then, for given $\lambda \ge 0, b\in Y$, $A:X\rightarrow Y$ and $\Phi :X\rightarrow Z$ linear, we define for all $x\in X$,

$$\begin{aligned} f(x)=\left\Vert Ax-b\right\Vert _Y^2+\lambda \left\Vert \Phi x\right\Vert _p. \end{aligned}$$

### Theorem

For all $0<p\le \infty$, $\lambda \ge 0$ there exists $x^\#\in X$ such that

$$\begin{aligned} \inf _{x\in X}f(x)=f(x^\#). \end{aligned}$$

In particular, the generalized LASSO problem (57) has at least one solution.

### Proof

Clearly, the function $f(x)=\left\Vert b\right\Vert _Y^2+\lambda \left\Vert \Phi x\right\Vert _p$ attains its minimum in $x^\#=0$. Hence, we may assume that

$$\begin{aligned} {{\,\textrm{Im}\,}}(A)\ne \{0\}. \end{aligned}$$

Since *Image*(*A*) is a vector subspace of *Y*, for all $b\in Y$ there exists a unique $y^\#\in Image(A)$ such that $\inf _{y\in Y}\left\Vert y-b\right\Vert _Y^2=\left\Vert y^\#-b\right\Vert _Y^2$. By definition, $y^\#=Ax^\#$ for some $x^\#\in X$. Hence,

$$\begin{aligned} \inf _{x\in X}\left\Vert Ax-b\right\Vert _Y^2=\inf _{y\in Image(A)}\left\Vert y-b\right\Vert _Y^2=\min _{y\in Y}\left\Vert y-b\right\Vert _2^2=\left\Vert Ax^\#-b\right\Vert _Y^2 \end{aligned}$$

and the assertion follows also for the case in which $\lambda =0$ or $Image(B)=\{0\}$. We will thereby assume that ${{\,\textrm{Im}\,}}(A)\ne \{0\}$, ${{\,\textrm{Im}\,}}(B)\ne \{0\}$ and $\lambda >0$. Let $L:=\text {ker}(A)\cap \text {ker}(B)=\{x\in X: Ax=0, \Phi x=0\}$ and denote the closed ball of *X* of center 0 and radius $r>0$ by $B_X(0,r)=\{x\in X: \left\Vert x\right\Vert _X\le r\}$. The rest of the proof is devided into three graded steps.

**Step 1.** **We prove that if** $L=\{0\}$, **then** $\lim _{\left\Vert x\right\Vert _X\rightarrow +\infty }f(x)=+\infty$.

By convexity of $\left\Vert \cdot \right\Vert _Y$,

$$\begin{aligned} \left\Vert y_1\right\Vert _Y^2\le 2(\left\Vert y_1-y_2\right\Vert _Y^2+\left\Vert y_2\right\Vert ^2_Y) \end{aligned}$$

for all $y_1,y_2\in Y$. Therefore,

$$\begin{aligned} \left\Vert Ax-b\right\Vert _2^2\ge \frac{1}{2}\left\Vert Ax\right\Vert _Y^2-\left\Vert b\right\Vert _Y^2, \end{aligned}$$

so that:

$$\begin{aligned} \left\Vert Ax-b\right\Vert _Y^2+\lambda \left\Vert \Phi x\right\Vert _p\ge \frac{1}{2}\left\Vert Ax\right\Vert _Y^2+\lambda \left\Vert \Phi x\right\Vert _p-\left\Vert b\right\Vert _Y^2. \end{aligned}$$

Let

$$\begin{aligned} \mathbb {S}_X:=\{x\in X:\left\Vert x\right\Vert _X=1\} \end{aligned}$$

denote the unit sphere of *X* and set $\eta :=\min _{x\in \mathbb {S}_X}\frac{1}{2}\left\Vert Ax\right\Vert _Y^2+\lambda \left\Vert \Phi x\right\Vert _p$. If $\eta =0$, then,

$$\begin{aligned} \min _{x\in \mathbb {S}_X}\frac{1}{2}\left\Vert Ax\right\Vert _Y^2+\lambda \left\Vert \Phi x\right\Vert _p=0, \end{aligned}$$

together with the assumptions on ${{\,\textrm{Im}\,}}(A)$, ${{\,\textrm{Im}\,}}(B)$ and $\lambda$, yields to the existence of $x^\#\in \mathbb {S}_X$ such that $\frac{1}{2}\left\Vert Ax^\#\right\Vert _Y^2+\lambda \left\Vert \Phi x^\#\right\Vert _p=0$. But $\left\Vert \cdot \right\Vert _Y$ and $\left\Vert \cdot \right\Vert _p$ are (quasi-)norms, so $x^\#=0\notin \mathbb {S}_X$. This is a contradiction. Hence, $\eta >0$.

Next, for $\left\Vert x\right\Vert _X>1$, we have:

$$\begin{aligned} \frac{1}{2}\left\Vert Ax\right\Vert _Y^2+\lambda \left\Vert \phi x\right\Vert _p-\left\Vert b\right\Vert _Y^2&=\frac{1}{2}\left\Vert x\right\Vert _X^2\left\Vert A\frac{x}{\left\Vert x\right\Vert _X}\right\Vert _Y^2+\lambda \left\Vert x\right\Vert _X\left\Vert \Phi \frac{x}{\left\Vert x\right\Vert _X}\right\Vert _p-\left\Vert b\right\Vert _Y^2\\&>\left\Vert x\right\Vert _X\left( \frac{1}{2}\left\Vert A\frac{x}{\left\Vert x\right\Vert _X}\right\Vert _Y^2+\lambda \left\Vert B\frac{x}{\left\Vert x\right\Vert _X}\right\Vert _p\right) -\left\Vert b\right\Vert _Y^2\\&\ge \eta \left\Vert x\right\Vert _X-\left\Vert b\right\Vert _Y^2. \end{aligned}$$

Therefore, for all $x\in X$ such that $\left\Vert x\right\Vert _X>1$,

$$\begin{aligned} f(x)=\left\Vert Ax-b\right\Vert _Y^2+\lambda \left\Vert \Phi x\right\Vert _p>\eta \left\Vert x\right\Vert _X+\left\Vert b\right\Vert _Y^2 \end{aligned}$$

and the assertion follows, since $\eta >0$ implies that the right hand-side goes to $+\infty$ as $\left\Vert x\right\Vert _X\rightarrow +\infty$.

**Step 2.** **We prove the assertion for** $L=\{0\}$.

Let $m:=\inf _{x\in X}f(x)$. By Step 1, there exist $R>0$ such that $f(x)>m+1$ for $\left\Vert x\right\Vert _X>R$. $B_X(0,R)$ is compact and convex, and $\inf _{x\in X}f(x)=\inf _{x\in B_X(0,R)}f(x)$ by definition of *R*. Let $(x_j)_j\subseteq B_X(0,R)$ be a minimizing sequence. By compactness, it admits a converging subsequence and, without loss of generality, we may assume that $\lim _{j\rightarrow +\infty }x_j=x^\#\in B_X(0,R)$. By continuity, $f(x^\#)=\lim _{j\rightarrow +\infty }f(x_j)=m$.

**Step 3.** **We prove the assertion for** $L\ne \{0\}$.

Recall that $X=L\oplus L^\perp$, where the orthogonality is defined with respect to the inner product $\langle \cdot ,\cdot \rangle _X$. By definition of direct sum, for all $x\in X$ there exist unique $x_1\in L$ and $x_2\in L^\perp$ such that $x=x_1+x_2$. Observe that since $x_1\in L$,

$$\begin{aligned} f(x)=\left\Vert Ax_2-b\right\Vert _Y^2+\lambda \left\Vert \Phi x_2\right\Vert _p=f(x_2). \end{aligned}$$

In particular,

$$\begin{aligned} \inf _{x\in X}f(x)=\inf _{x\in L^\perp }f(x). \end{aligned}$$

The restrictions of *A* and $\Phi$ to $L^\perp$ are linear mappings between vector spaces. We denote them with $A|_{L^\perp }L^\perp \rightarrow Y$ and $\Phi |_{L^\perp }:L^\perp \rightarrow Z$ respectively and set $f|_{L^\perp }:L^\perp \rightarrow Y$ as the restriction of *f* to $L^\perp$. Obviously,

$$\begin{aligned} f|_{L^\perp }(x)=\left\Vert A|_{L^\perp }x-b\right\Vert _Y^2+\lambda \left\Vert \Phi |_{L^\perp }x\right\Vert _p=f(x) \end{aligned}$$

for all $x\in L^\perp$, so that:

$$\begin{aligned} \inf _{x\in X}f(x)=\inf _{x\in L^\perp }f(x)=\inf _{x\in L^\perp }f|_{L^\perp }(x). \end{aligned}$$

Obviously,

$$\begin{aligned} L^\perp :=\ker (A|_{L^\perp })\cap \ker (\Phi |_{L^\perp })=\ker (A)\cap \ker (B)\cap L^\perp =L\cap L^\perp =\{0\}. \end{aligned}$$

Therefore, by Step 2, it follows that there exists $x^\#\in L^\perp$ such that:

$$\begin{aligned} \inf _{x\in L^\perp }f|_{L^\perp }(x)=f|_{L^\perp }(x^\#). \end{aligned}$$

This implies that:

$$\begin{aligned} \inf _{x\in X}f(x)=f|_{L^\perp }(x^\#)=f(x^\#), \end{aligned}$$

since $$x^\#\in L^\perp $$. $$\square $$
